# Metagenome-genome-wide association studies reveal human genetic impact on the oral microbiome

**DOI:** 10.1038/s41421-021-00356-0

**Published:** 2021-12-07

**Authors:** Xiaomin Liu, Xin Tong, Jie Zhu, Liu Tian, Zhuye Jie, Yuanqiang Zou, Xiaoqian Lin, Hewei Liang, Wenxi Li, Yanmei Ju, Youwen Qin, Leying Zou, Haorong Lu, Shida Zhu, Xin Jin, Xun Xu, Huanming Yang, Jian Wang, Yang Zong, Weibin Liu, Yong Hou, Huijue Jia, Tao Zhang

**Affiliations:** 1 BGI-Shenzhen, Shenzhen, Guangdong, China; 2 College of Life Sciences, University of Chinese Academy of Sciences, Beijing, China; 3 Department of Biology, University of Copenhagen, Universitetsparken 13, Copenhagen, Denmark; 4 Qingdao-Europe Advanced Institute for Life Sciences, BGI-Shenzhen, Qingdao, Shandong, China; 5 School of Bioscience and Biotechnology, South China University of Technology, Guangzhou, Guangdong, China; 6 China National Genebank, BGI-Shenzhen, Shenzhen, Guangdong, China; 7 James D. Watson Institute of Genome Sciences, Hangzhou, Zhejiang, China; 8 Shenzhen Key Laboratory of Human Commensal Microorganisms and Health Research, BGI-Shenzhen, Shenzhen, Guangdong, China

**Keywords:** Molecular biology, Genome-wide association studies

## Abstract

The oral microbiota contains billions of microbial cells, which could contribute to diseases in many body sites. Challenged by eating, drinking, and dental hygiene on a daily basis, the oral microbiota is regarded as highly dynamic. Here, we report significant human genomic associations with the oral metagenome from more than 1915 individuals, for both the tongue dorsum (*n* = 2017) and saliva (*n* = 1915). We identified five genetic loci associated with oral microbiota at study-wide significance (*p* < 3.16 × 10^−11^). Four of the five associations were well replicated in an independent cohort of 1439 individuals: rs1196764 at *APPL2* with *Prevotella jejuni*, *Oribacterium uSGB 3339* and *Solobacterium uSGB 315*; rs3775944 at the serum uric acid transporter *SLC2A9* with *Oribacterium uSGB 1215*, *Oribacterium uSGB 489* and *Lachnoanaerobaculum umeaense*; rs4911713 near *OR11H1* with species *F0422 uSGB 392;* and rs36186689 at *LOC105371703* with *Eggerthia*. Further analyses confirmed 84% (386/455 for tongue dorsum) and 85% (391/466 for saliva) of host genome-microbiome associations including six genome-wide significant associations mutually validated between the two niches. As many of the oral microbiome-associated genetic variants lie near miRNA genes, we tentatively validated the potential of host miRNAs to modulate the growth of specific oral bacteria. Human genetics accounted for at least 10% of oral microbiome compositions between individuals. Machine learning models showed that polygenetic risk scores dominated over oral microbiome in predicting risk of dental diseases such as dental calculus and gingival bleeding. These findings indicate that human genetic differences are one explanation for a stable or recurrent oral microbiome in each individual.

## Introduction

A healthy individual swallows 1–1.5 L of saliva every day^1^, and its residing microbes could colonize the gut of susceptible individuals^2–4^. Oral metagenomic shotgun sequencing data has been available from the Human Microbiome Project (HMP)^5^, for rheumatoid arthritis^6^ and colorectal cancer^3^. Other diseases such as liver cirrhosis, atherosclerotic cardiovascular diseases, type 2 diabetes and colorectal cancer studied by metagenome-wide association studies (MWAS) using gut microbiome data also indicated potential contribution of the oral microbiome to disease etiology^2,7–10^.

Controversy over whether human genetics or environments dominate the fecal microbiome is being clarified by an increasing number of studies^11–16^. The strongest signal in cohorts of European ancestry is the association between *LCT1* and *Bifidobacterium*, explained by the metabolism of lactose by the commensal bacterium. These large-scale genome-wide association studies have mainly focused on the fecal microbiome; however, the influence of host genetics on the composition and stability of the oral microbiome is still poorly understood. Several studies based on 16 S rRNA amplicon sequencing and microarrays have reported that human oral microbiota are influenced by both host genetics and environmental factors^17–20^. Only two studies have identified limited human genes that affected oral microbial communities. One study identified that *IMMPL2* on chromosome 7 and *INHBA-AS1* on chromosome 12 could influence microbiome phenotypes^18^. The other study reported a gene copy number (CN) of the *AMY1* locus correlated with the oral and gut microbiome composition and function^21^. These two studies used 16 S rRNA amplicon sequencing for a small number of samples. Therefore, the influence of human genes on the composition of the oral microbiome and genetic stability between different oral niches are still poorly understood.

Here, we presented the first large-scale metagenome-genome-wide association studies (mgGWAS) for 2017 tongue dorsum samples and 1915 salivary samples from a cohort of 2984 healthy Chinese individuals with high-depth whole-genome sequencing data. We further validated the identified associations in an independent replication cohort of 1494 individuals with also metagenomic sequencing data and relatively low-depth whole-genome sequencing data. A large number of concordant associations were identified between genetic loci and the tongue dorsum and salivary microbiomes. The effects of environmental factors and host genes on oral microbiome composition were investigated. Host genetics explained more variances of microbiome composition than environmental factors. The findings underscore the value of mgGWAS for in situ microbial samples, instead of focusing on feces.

## Results

### The oral microbiome according to metagenomically assembled microbial genomes

The 4D-SZ cohort (multi-omics, with more data to come, from Shenzhen, China) at present have high-depth whole-genome sequencing data from 2984 individuals (mean depth of 33×, ranging from 15× to 78×, Supplementary Table S1 and Fig. S1). Among these, 2017 individuals had matched tongue dorsum and 1915 individuals had matched salivary samples for mgGWAS analyses, with no population stratification (Supplementary Fig. S2).

Shotgun metagenome sequencing was performed for the 3,932 oral samples, with an average sequencing data of 19.18 ± 7.90 Gb for 2017 tongue dorsum and 13.64 ± 2.91 Gb for 1915 salivary samples (Supplementary Table S1 and Fig. S1). The microbiome composition was determined according to alignment to a total of 56,213 metagenome-assembled genomes (MAGs) that have been organized into 3589 species-level clusters (SGBs) together with existing genomes, of which 40% (1441/3589) was specific in this cohort^22^. Both the tongue dorsum and the salivary samples contained the phyla *Bacteroidetes* (relative abundance of 37.2% ± 11.3% for tongue dorsum and 40.1% ± 10.2% for saliva, respectively), *Proteobacteria* (30.1% ± 16.5% and 30.6% ± 13.1%, respectively), *Firmicutes* (20.5% ± 8.2% and 17.7% ± 6.7%, respectively), *Actinobacteria* (4.3% ± 3.4% and 2.6% ± 2.0%, respectively), *Fusobacteria* (4.0% ± 1.9% and 3.3% ± 1.4%, respectively), *Patescibacteria* (in Candidate Phyla Radiation, CPR, 2.5% ± 1.6% and 3.1% ± 1.6%, respectively), and *Campylobacterota* (1.1% ± 0.9% and 1.3% ± 0.8%, respectively) (Supplementary Fig. S3a, b). These seven phyla cover between 99.7% (tongue dorsum) and 98.7% (saliva) of the whole community, indicating that the two oral sites share a common core microbiota. Consistent with HMP results using 16 S rRNA gene amplicon sequencing^23^, the salivary samples presented a higher alpha diversity than tongue dorsum samples (mean Shannon index of 6.476 vs 6.228; Wilcoxon rank-sum test *p* < 2.2 × 10^−16^; Supplementary Fig. S3c). The microbiome compositions calculated by beta-diversity based on genus-level Bray–Curtis dissimilarity slightly differed (explained variance *R*^2^ = 0.055, *p* < 0.001 in permutational multivariate analysis of variance (PERMANOVA) test; Supplementary Fig. S3d).

### Host genetic variants strongly associated with the tongue dorsum microbiome

**Fig. 1:**
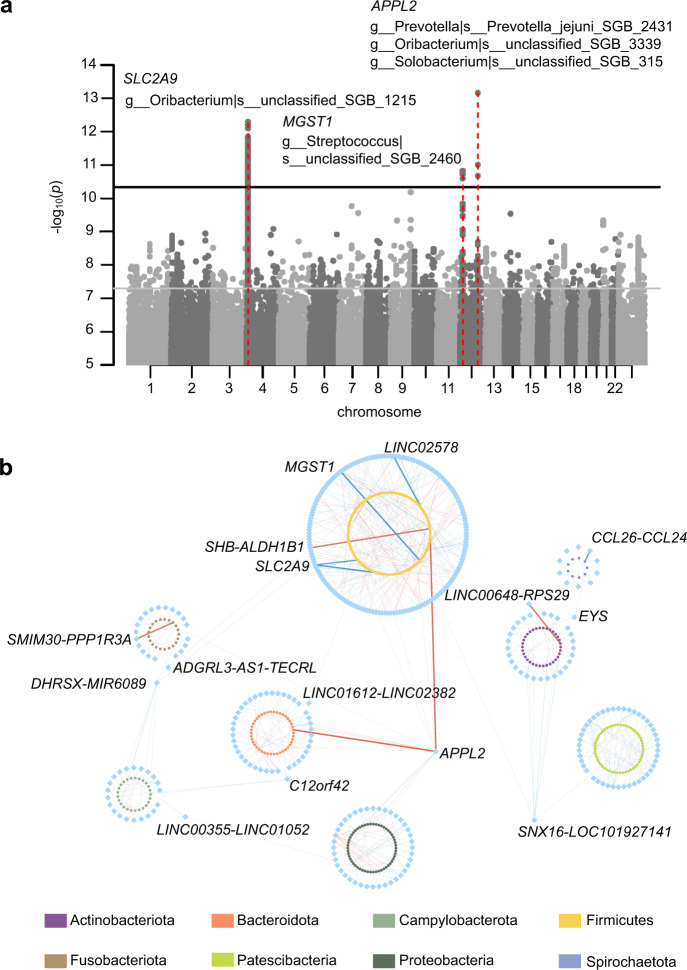
Host genetic signals associated with the tongue dorsum microbiome. **a** Manhattan plot shows the genetic variants associated with the tongue dorsum microbial taxa. The horizontal gray and black lines represent the genome-wide (*p* = 5 × 10^−8^) and study-wide (*p* = 3.16 × 10^−11^ for 1583 independent mgGWAS tests) significance levels, respectively. Three loci that were associated with the tongue dorsum microbiome and reached study-wide significance were marked in red. Their located genes and associated microbial taxa with *p* values of < 3.16 × 10^−11^ were also listed. **b** Network representation of the 455 gene-microbiome associations identified in the tongue dorsum mgGWAS at the genome-wide significance. Each node represents either a gene (blue diamonds) or a microbial taxon (circles with different colors according to phylum). Each edge is an association between one gene and one microbial taxon. The bold edge represented study-wide significant associations as shown in **a**. The genes that linked to at least two different microbial taxa from different phyla were also listed.

With this so far, the largest cohort of the whole genome and whole metagenome data, we first performed mgGWAS on the tongue dorsum microbiome. With the 1583 independent tongue dorsum microbial taxa (*r*^2^ < 0.8 from 3177 taxa total using a greedy algorithm, Materials and methods), and 10 million human genetic variants (minor allele frequency (MAF) ≥ 0.5%), 455 independent associations involving 340 independent loci (distance < 1 Mb and *r*^2^ < 0.2) and 385 independent taxa reached genome-wide significance (*p* < 5 × 10^−8^). With a more conservative Bonferroni-corrected study-wide significant *p* value of 3.16 × 10^−11^ (= 5 × 10^−8^/1583), we identified three genomic loci, namely *APPL2*, *SLC2A9*, and *MGST1*, associated with five tongue dorsum microbial features involving 112 SNP-taxon associations (Fig. 1a). These associations showed remarkable evidence of polygenicity and pleiotropy (Fig. 1b). There was no evidence of any excess false positive rate in the GWAS analyses (genomic inflation factors λ_GC_ ranged from 0.981 to 1.023 with a median of 1.005; Supplementary Fig. S4a). All genome-wide significant associations were listed in Supplementary Table S2.

We also used a replication cohort to validate these associations. The replication cohort was comprised of 1494 individuals from multiple cities in China (also shotgun metagenomic sequencing for 1333 tongue dorsum samples to an average of 19.90 ± 7.73 Gb and 1299 salivary samples to an average of 13.66 ± 2.80 Gb, but about 9× whole-genome sequencing for the human genome; Supplementary Table S1 and Fig. S1). Among the 455 independent associations identified in the discovery cohort with *p* < 5 × 10^−8^, 33 were not available in the low-depth replication dataset. We were able to replicate 37 of the remaining 422 associations (replication rate: 8.77%) in the same effect direction of the minor allele (*p* < 0.05; Supplementary Table S2), which is much higher than expected by chance (average replication rate: 2.95%; *χ*^2^ = 12.74, df = 1, *p* = 3.6 × 10^−4^). Two of the three study-wide signals from the discovery cohort were well replicated: rs1196764 at *APPL2* with *Prevotella jejuni*, *Oribacterium uSGB 3339*, and *Solobacterium uSGB 315*; and rs3775944 at *SLC2A9* with *Oribacterium uSGB 1215*, *Oribacterium uSGB 489,* and *Lachnoanaerobaculum umeaense*.

**Fig. 2:**
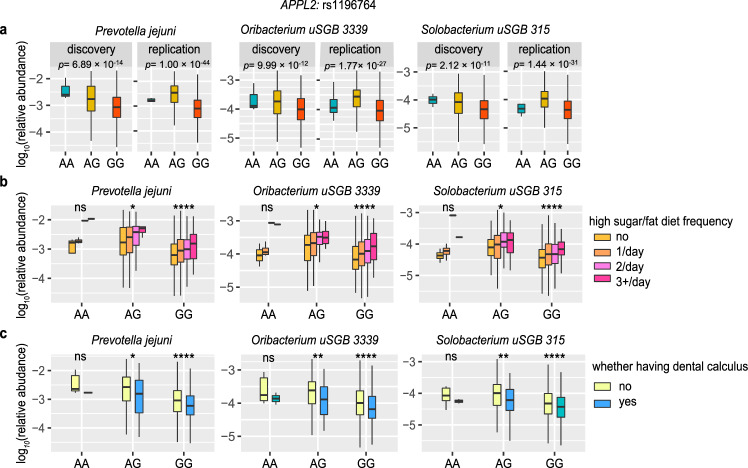
The links among human APPL2 locus (rs1196764), three tongue dorsum bacteria, and diet as well as the status of dental calculus. **a** The three panels presented the associations of *APPL2* variation with the three most significantly associated taxa: *Prevotella jejuni*, *Oribacterium uSGB 3339* and *Solobacterium uSGB 315*, which reached study-wide significance in both the discovery (*n* = 2017) and replication (*n* = 1333) cohorts. **b** Relative abundance of the three taxa across stratified groups of individuals according to *APPL2*: rs1196764 variation and self-reported high sugar/fat dietary frequency (colored by no intake, 1/day; 2/day; 3+/day, respectively). **c** Relative abundance of the three taxa across stratified groups of individuals according to *APPL2*: rs1196764 variation and whether having dental calculus (yellow: no and blue: yes). All statistical comparisons in **b** and **c** denote the *p* values of the Wilcoxon rank test on the total 3350 individuals with log-transformed relative abundances. The significant code of *p* values thresholds are shown: ns, *p* > 0.05; *p ≤ 0.05; **p ≤ 0.01; ***p ≤ 0.001; ****p ≤ 0.0001.

The strongest association identified by tongue dorsum mgGWAS was on rs1196764 located in the *APPL2* locus, with minor allele A (MAF = 0.06) positively associated with abundances of three species, namely *Prevotella jejuni* (Fig. 2a; *p*_discovery_ = 6.89 × 10^−14^; *p*_replication_ = 1.00 × 10^−44^), *Oribacterium unclassified SGB (uSGB) 3339* (*p*_discovery_ = 9.99 × 10^−12^; *p*_replication_ = 1.77 × 10^−27^), and *Solobacterium uSGB 315* (an anaerobic gram-positive bacterium associated with colorectal cancer^24^; *p*_discovery_ = 2.12 × 10^−11^; *p*_replication_ = 1.44 × 10^−31^). *APPL2* encoded a multifunctional adapter protein that binds to various membrane receptors, nuclear factors and signaling proteins to regulate many processes, such as cell proliferation, immune response, endosomal trafficking and cell metabolism. *APPL2*-associated three taxa all positively correlated with high sugar/fat dietary frequency (Fig. 2b; Spearman *p* = 3.18 × 10^−6^ for *Prevotella jejuni, p* = 1.33 × 10^−5^ *for Oribacterium uSGB 3339* and *p* = 6.43 × 10^−9^ for *Solobacterium uSGB 315*) when checking the correlation between oral taxa and phenotypic traits in this cohort, in line with the role of *APPL2* in controlling glucose-stimulated insulin secretion^25^. Appl2 protein had been reported to play a negative regulatory role in inflammation^26^. Its associated three taxa also correlated with decreasing risk of dental calculus and gingival bleeding (Fig. 2c; Spearman *p* < 0.001), thereby supporting a link between genetic variation in the *APPL2* gene, immune response, and the abundance of these taxa.

**Fig. 3:**
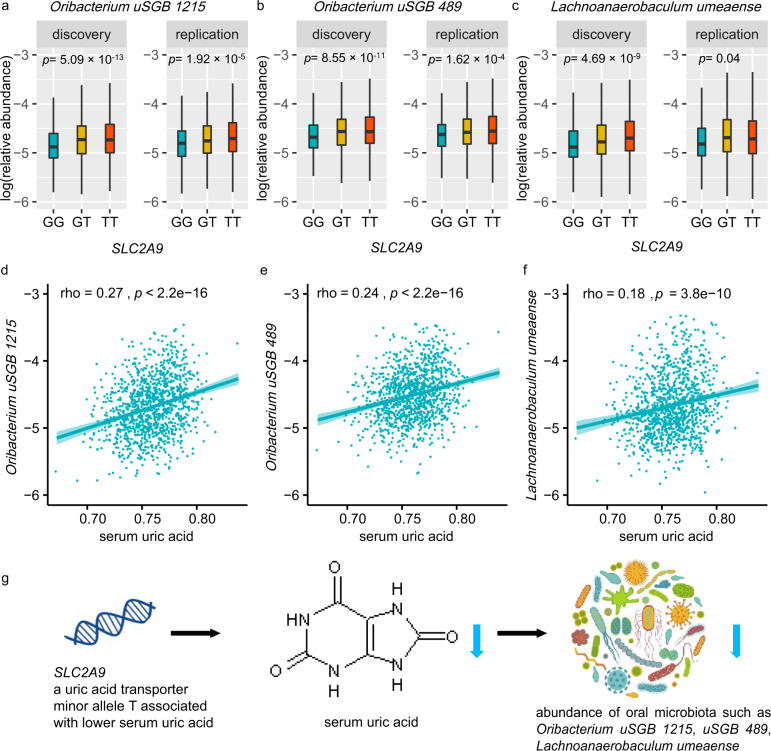
Interaction of human SLC2A9 locus (rs3775944), serum uric acid, and three tongue dorsum bacteria. **a–c** The three panels present the associations of *SLC2A9* variation with microbial abundances of the three most significantly associated taxa: *Oribacterium uSGB 1215*, *Oribacterium uSGB 489*, and *L. umeaense*), which reached study-wide significance in the discovery (*n* = 2017) cohort and were well replicated in the replication (*n* = 1333) cohort. **d–f** Correlations between three tongue dorsum bacteria and serum uric acid. Spearman’s correlation coefficient (rho) and *p* value were shown. **g** Schematic representation of the interaction among *SLC2A9* locus (rs3775944), serum uric acid, and three tongue dorsum bacteria: *SLC2A9* as a uric acid transporter, its minor allele T of SNP rs3775944 associated with lower serum uric acid, and genetic predisposition to lower serum uric acid level is associated with lower abundance of *Oribacterium uSGB 1215*, *Oribacterium uSGB 489*, and *Lachnoanaerobaculum umeaense*.

The second strongest association was on rs3775944, which is a perfect proxy for the exonic variant rs10939650 (*r*^2^ = 0.99) in *SLC2A9*. Minor allele G of rs3775944 (MAF = 0.49) in *SLC2A9* locus negatively correlated with *Oribacterium uSGB 1215* (Fig. 3a; *p*_discovery_ = 5.09 × 10^−13^; *p*_replication_ = 1.92 × 10^−5^), *Oribacterium uSGB 489* (Fig. 3b; *p*_discovery_ = 8.55 × 10^−11^; *p*_replication_ = 1.62 × 10^−4^), and *L. umeaens*e (Fig. 3c; *p*_discovery_ = 4.69 × 10^−9^; *p*_replication_ = 0.04)*. SLC2A9* is a urate transporter and *SLC2A9* polymorphisms have been reported associated with serum uric acid and urine uric acid concentration in multiple studies^27–29^. We also looked at these top loci in Biobank Japan (BBJ)^30,31^, and *SLC2A9* was correlated with lower serum uric acid concentration (Supplementary Fig. S5; *p* = 5.56 × 10^−184^), ischemic stroke (*p* = 1.73 × 10^−4^), urolithiasis (*p* = 2.02 × 10^−4^), and pulse pressure (*p* = 6.86 × 10^−4^). The negative associations of *SLC2A9* with serum uric acid concentration (*p* = 6.74 × 10^−6^) and urine pH (*p* = 8.75 × 10^−4^) were confirmed in this cohort (Supplementary Fig. S5). Interestingly, serum uric acid level highly correlated with *Oribacterium uSGB 1215* (Fig. 3d; Spearman rho = 0.27, *p* < 2.2 × 10^−16^), *Oribacterium uSGB 489* (Fig. 3e; *rho* = 0.24, *p* < 2.2 × 10^−16^), and *L. umeaense* (Fig. 3f; rho = 0.18, *p* = 3.8 × 10^−10^). These results presented a potential explanation for *SLC2A9* acting on three oral taxa through serum uric acid as intermedium (Fig. 3g). Likewise, the lipoprotein lipase (*LPL) gene* was a determinant of triglyceride concentration (Supplementary Fig. S5; *p* = 5.93 × 10^−56^), triglyceride concentration correlated with abundance of *Haemophilus D parainfluenzae A* (*p* = 7.70 × 10^−16^), and consistently *LPL* exhibited significant association with *Haemophilus D parainfluenzae A* (*p* = 1.59 × 10^−8^). These findings suggested that host genes may regulate oral microbiota by mediating their relevant metabolites.

Variants in *MGST1* (leading SNP rs7294985) were identified as the third strongest signal, with minor allele negatively associated with *Streptococcus uSGB 2460* (*p*_discovery_ = 1.50 × 10^−11^; *p*_replication_ = 0.92), followed by family Streptococcaceae and other eight *Streptococcus* SGBs, such as *S. infantis* and *S. pseudopneumoniae*, although not confirmed in the replication cohort. These variants were also positively associated with red blood cell count (*p* = 2.51 × 10^−5^) and asthma (*p* = 5.03 × 10^−5^) in Biobank Japan. Consistently, 84% (237/282) of the *Streptococcus spp*. were observed correlated with red blood cell count (*p* < 0.05), such as *S. mitis (p* = 1.99 × 10^−12^*)* and *S. pseudopneumoniae (p* = 4.51 × 10^−12^*)*. These results suggested that commensal *Streptococcus* species might utilize red blood cells as camouflage to avoid being engulfed by phagocytic immune cells in addition to the well-known group A Streptococcus (*S. pyogenes*)^32^. Our results also supported previous findings that *Streptococcus spp*. are often involved in diseases of the respiratory tracts such as asthma^33^.

In addition to the above three study-wide significant loci, other well replicated genome-wide significant associations included rs17070896 in *ADAMTS9* with *Simonsiella muelleri*, rs59134851 near *MST1L-MIR3675* with *Streptococcus anginosus* (playing important roles in respiratory infections^34^), chr22:41198300 in *EP300-AS1* with *Parvimonas micra* (potential pathogen of colorectal cancer^10^), rs34555647 near *MIR3622B-CCDC25* with *Selenomonas sputigena* (potential pathogen of periodontal diseases^35^). These replicated associations invited further investigation of the impacts of host–microbial interactions on disease.

### mgGWAS of the salivary microbiome confirm and extend human genetic contribution to the oral microbiome

**Fig. 4:**
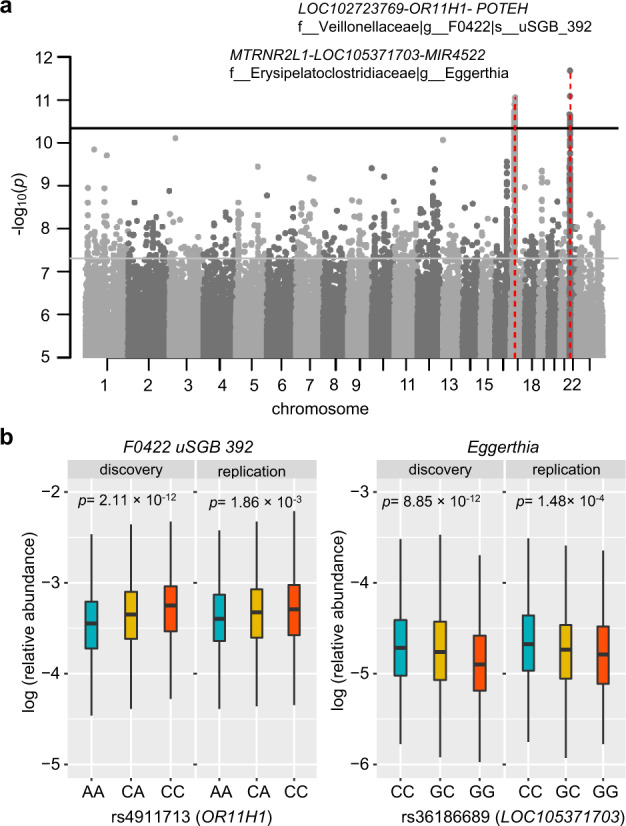
Host genetic signals associated with the salivary microbiome. **a** Manhattan plot shows the genetic variants associated with the salivary microbial taxa. The horizontal gray and black lines represent the genome-wide (*p* = 5 × 10^−8^) and study-wide (*p* = 2.97 × 10^−11^ for 1685 independent mgGWAS tests) significance levels, respectively. Two loci that were associated with the salivary microbiome and reached study-wide significance were marked in red. **b** Their located genes and associated microbial taxa with *p* values of < 2.97 × 10^−11^ were also listed.

The saliva may appear more dynamic than the tongue dorsum, and the microbiome composition involves multiple niches in the oral cavity^36^. We next tried mgGWAS analysis for the saliva microbiome. With the 1685 independent salivary microbial taxa (*r*^2^ < 0.8 from 3677 taxa total), and 10 million human genetic variants (MAF ≥ 0.5%) in discovery cohort, 466 independent associations involving 374 independent loci (*r*^2^ < 0.2) reached genome-wide significance (*p* < 5 × 10^−8^). With a more conservative Bonferroni-corrected study-wide significant *p* value of 2.97 × 10^−11^ (= 5 × 10^−8^/1685), two study-wide significant independent loci were identified (Fig. 4a). Similar to tongue dorsum mgGWAS analyses, the genomic inflation factors of these salivary mgGWAS tests showed no inflation (λ_GC_ ranged from 0.978 to 1.022 with a median of 1.002; Supplementary Fig. S4b). All genome-wide significant associations were listed in Supplementary Table S3.

As for validation, we were able to replicate 28 of the remaining 443 associations in the same effect direction of the minor allele (*p* < 0.05), given that 23 of the 466 independent associations identified in the discovery cohort with *p* < 5 × 10^−8^ were not available in the low-depth replication dataset (Supplementary Table S3). The fraction of replicated associations (6.32%) was much higher than expected by chance (average replication rate: 2.71%; *χ*^2^ = 5.891, df = 1, *p* = 0.015). Two study-wide significant signals identified by this saliva mgGWAS were both well replicated (Fig. 4b). One genetic locus, spanning three genes *LOC102723769*, *OR11H1*, and *POTEH*, associated with species *F0422 uSGB 392* belonging to family Veillonellaceae (leading SNP rs4911713; *p*_discovery_ = 2.11 × 10^−12^; *p*_replication_ = 1.86 × 10^−3^). *F0422 uSGB 392* negatively correlated with concentrations of serum amino acids such as cysteine and glycine and levels of blood microelements such as magnesium and lead, as well as serum testosterone levels and mental distress (impatience or tension) (Supplementary Fig. S6). The locus was consistently associated with mental distress (impatience) and serum testosterone level (*p* < 0.05), while searching GWAS summary statistics from Biobank Japan and this study. The other locus, *MTRNR2L1-LOC105371703-MIR4522*, is associated with the genus *Eggerthia* (leading SNP rs36186689; *p*_discovery_ = 8.85 × 10^−12^; *p*_replication_ = 1.48 × 10^−4^). *Eggerthia* was most positively associated with frequency of gingival bleeding, dental calculus, frequency of tooth pain, and dental periodontitis, but negatively linked to serum hormones such as cortisone, aldosterone, and testosterone (Supplementary Fig. S7). The locus was most associated with glaucoma and serum creatine level while searching GWAS summary statistics from Biobank Japan and this study. Notably, the two loci both regulated the expression of genes in the testis or brain cerebellar hemisphere (*p* < 10^−5^) when searching in the GTEx^37^ database, and their associated oral taxa both consistently correlated with serum testosterone levels and mental distress (impatience)(Supplementary Figs. S6 and S7). In addition, we found four loci associated with both the salivary microbiome and metabolic traits or diseases at genome-wide significance: *DPEP2/NFATC3* that associated with species *Lancefieldella sp000564995* was linked to high-density lipoprotein cholesterol (HDLC); *PDXDC2P-NPIPB14P* associated with species *Centipeda sp000468035* linked to thyroid abnormality; *LARP1* associated with species *Aggregatibacter kilianii* linked to mean corpuscular hemoglobin; *SMARCA1* associated with species *Veillonella parvula* linked to pharyngeal mucosal congestion (PMC) (Supplementary Fig. S8). These results again supported the “host genes—blood metabolites—oral microbiota” interactive axis in the human body.

**Fig. 5:**
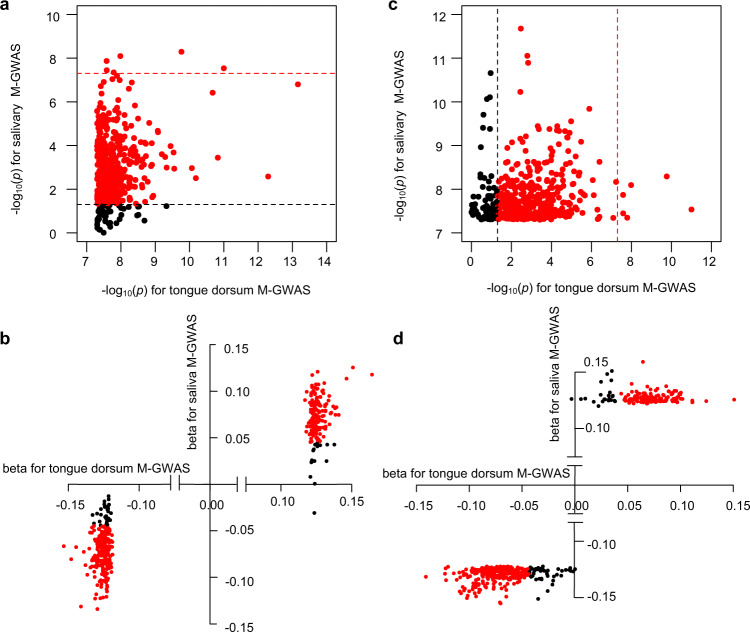
Comparisons of mgGWAS associations between tongue dorsum and saliva samples. **a** *p*-values comparisons of the 345 and 374 independent loci associated with tongue dorsum and salivary microbiome (*p* < 5 × 10^−8^), respectively. **b** β-values comparisons of the 345 and 374 independent loci associated with tongue dorsum and salivary microbiome (*p* < 5 × 10^−8^*)*, respectively. In **a** and **b**, the six genome-wide significant loci shared by tongue dorsum and salivary microbiome were listed. The dots marked red and black represented shared associations by both (*p* < 5 × 10^−8^ in one niche and *p* < 0.05 in the other niche in the same direction of the minor allele) and specific associations (*p* < 5 × 10^−8^ in one niche but *p* > 0.05 in the other niche or the opposite direction of the minor allele) in one niche, respectively.

Among 455 and 466 independent associations identified for tongue dorsum and salivary microbiome (*p* < 5 × 10^−8^), respectively, six were shared between them (Fig. 5): *APPL2* associated with *Oribacterium uSGB 3339*, *LOC105374972-NRSN1* associated with *Lancefieldella uSGB 2019*; *CCL26-CCL24* associated with *Treponema B uSGB 706*; *RALGPS2* associated with *Scardovia wiggsiae*; *KRT16P1-LGALS9C* associated with *Patescibacteria uSGB 2650*; and *RTTN-SOCS6* associated with *Firmicutes uSGB 1705*. More specifically, among the 455 independent and genome-wide significant variants-taxa associations for the tongue dorsum samples, 386 associations (85%) were replicated with *p* < 0.05 in the same effect direction of minor allele for the salivary samples (Fig. 5a, b). For example, *SLC2A9*, a determinant of low uric acid (UA) concentration, showed the strongest association with SGBs belonging to *Oribacterium* (*p* = 5.09 × 10^−13^) and *Lachnoanaerobaculum* (*p* = 4.69 × 10^−9^) in tongue dorsum samples, and also a relative low association with that of *Oribacterium* (*p* = 0.001) and *Lachnoanaerobaculum* (*p* = 1.0 × 10^−4^) in salivary samples. Among the 466 independent and genome-wide significant variants-taxa associations for saliva samples, 391 associations (84%) also were replicated with *p* < 0.05 in the same effect direction of minor allele for the tongue dorsum (Fig. 5c, d), although the top two study-wide significant loci for saliva did not reach suggestive significance in tongue dorsum samples (*p* > 1 × 10^−5^). Our mgGWAS of the salivary microbiome further confirm and extend human genetic contribution to the oral microbiome. These results suggested tongue and salivary microbiome as niches in one oral cavity shared a high level of host genetic similarity in the coevolution process.

### Gene set enrichment analysis for oral mgGWAS signals

To explore the potential functions of the identified mgGWAS signals for tongue dorsum and salivary, we annotated the genetic associations and performed functional mapping and gene sets enrichment analysis with the DAVID^38^ and FUMA^39^ platform (Materials and methods), followed by disease enrichment and tissue expression analysis. These mgGWAS analyses returned 221 and 261 genes (< 20 Kb for associated genetic loci) for tongue dorsum and salivary microbiome, respectively. Functional mapping of their separately related genes in the DAVID database suggested that tongue dorsum associated host genes mainly enriched in phosphatidylinositol-related pathways including phosphatidylinositol signaling system, biosynthesis, dephosphorylation, and phosphatidylinositol-3,4,5-trisphosphate 5-phosphatase activity, and Ca^2+^ pathway including calcium ion binding, calcium channel regulator activity, and voltage-gated calcium channel activity (Supplementary Table S4). The phosphatidylinositol signaling system has been reported to be higher in the gut microbiota of centenarians^40^ and consistently decreased in saliva microbiota of RA patients^41^. Saliva-associated host genes were mainly enriched in cardiomyopathy including arrhythmogenic right ventricular-, hypertrophic- and dilated cardiomyopathy, glycerophospholipid metabolism, and choline metabolism in cancer (Supplementary Table S5).

The GAD_Disease (Genetic Association Disease Database) segment analysis in DAVID showed that both tongue dorsum and saliva mgGWAS signals were enriched in cardiometabolic diseases and traits such as tobacco use disorder, myocardial infarction, triglycerides, blood pressure, lipoproteins, coronary artery disease, and nervous system diseases such as schizophrenia, bipolar disorder, psychiatric disorders, and Parkinson’s disease (Supplementary Tables S4 and S5). Positional mapping in the GWAS catalog using the FUMA tool showed similar diseases enriched results with that of using the GAD catalog in DAVID. Genotype-Tissue Expression (GTEx) analysis on saliva microbiome-associated host genes exhibited enrichment for genes expressed in the brain (anterior cingulate cortex BA24 and substantia nigra) and cells of EBV-transformed lymphocytes (Supplementary Fig. S9).

### Potential modulation of oral bacteria by host miRNA

As many of the human genetic associations identified in the above mgGWAS analysis lay nearby miRNA genes, we next investigated the potential of miRNAs to modulate the growth of specific oral bacteria. *Streptococcus spp*. were the common taxa in the oral cavity and our mgGWAS showed that rs569277522 near *MIR4693* and rs2891896 near *MIR3977* associated with abundances of *S. oralis* (*p* = 4.33 × 10^−8^) and *S. infantis* (*p* = 1.254 × 10^−8^), respectively. Next, we investigated whether miR-4693 could regulate *S. oralis* and miR-3977 could regulate *S. infantis*. We blasted the 5p strand of miR-4693 (miR-4693-5p) sequence against the whole-genome sequence of *S. oralis* and found that eleven genes were predicted to be targeted by miR-4693-5p, of which four genes (locus tags: EL140_RS07695, EL140_RS00690, EL140_RS00965, and EL140_RS07695) were 16 S ribosomal RNA genes and were the most probable targets with minimum mfe (minimum free energy) of –28.4 kcal/mol (Supplementary Fig. S10a). We then cultured *S. oralis* in the presence of synthetic miR-4693-5p or not and found that miR-4693-5p inhibited the growth of *S. oralis* (Supplementary Fig. S10b). Likewise, four genes (locus tags: HMPREF9423_RS06630, HMPREF9423_RS03895, HMPREF9423_RS07625, and HMPREF9423_RS09390) from *S. infantis* genome were predicted to be targeted by miR-3977 and miR-3977 inhibited the growth of *S. infantis* (Supplementary Fig. S10c, d). These gene products were often on the bacterial ribosome, consistent with the effects on growth curves. Thus, similar to previous findings that host fecal miRNA is able to regulate bacterial growth by targeting specific bacterial genes^42,43^, our mgGWAS results here suggest that human miRNAs may specifically regulate the abundances of oral bacteria.

### Host genetics influence oral microbiome more than environments

We first investigated the contribution of the host environmental factors to oral microbiome β-diversity (based on genus-level Bray–Curtis dissimilarities), by using host metadata including age, gender, BMI, diets, lifestyles, drugs use, and health status questions, as well as blood measurements. We selected 340 independent variables out of the total 423 environmental factors for association analysis (correlation *r*^2^ < 0.6). A total of 35 and 53 host environmental factors were significantly associated with β-diversity (BH-adjusted FDR < 0.05) for tongue dorsum and salivary samples, respectively, via PERMANOVA analysis (Supplementary Fig. S11 and Tables S6, S7). Of these, high sugar and high-fat food frequency and dental calculus were among the strongest explanatory factors for both tongue dorsum and salivary microbial compositions. A high sugar diet increased the abundance of some specific bacteria such as *Streptococcus mutants* that metabolized sugar to acids and caused dental caries. In this cohort, high sugar and high-fat food frequency significantly increased the abundances of *Gemella haemolysans* (*β* = 0.21; *p* = 2.92 × 10^−19^) and *Streptococcus parasanguinis* (*β* = 0.18; *p* = 7.56 × 10^−16^) in salivary samples. In addition, gender, serum metabolites such as glutamic acid, cystine, and testosterone, as well as geographic location (residing in northern or southern China), all showed strong effects on the oral microbiome composition. In total, 35 and 53 host environmental factors were able to infer 6.36% and 7.78% of the variance of microbiome β-diversity for tongue dorsum and salivary samples, respectively. When calculating the cumulative explained variance of β-diversity by using all the independent environmental variables, we found that 12.85% and 15.54% of the variance can be explained for tongue dorsum and salivary samples, respectively.

We next evaluated the effect of host genetics on oral microbiome compositions. We performed association analysis for *α*-diversity and *β*-diversity using 10 million genetic variants (MAF ≥ 0.5%). Six genome-wide significant loci were identified for *α*-diversity of the oral microbiome (Supplementary Table S8). Four loci, *NFIB*, *LINC02578*, *LOC105373105*, and *EIF3E*, were associated with *α*-diversity of tongue dorsum samples. Two loci, *SLC25A42* and *LINC02225*, were associated with the *α*-diversity of salivary samples. In the association analysis between genetic variation and microbiome β-diversity, we found one locus for tongue dorsum samples and one locus for salivary samples with marginal genome-wide significance (*p* < 5 × 10^−8^; Supplementary Fig. S12), respectively. One SNP, rs545425011 located in *DNAJC12* was associated with the microbial composition of the tongue dorsum (*p* = 1.07 × 10^−8^). When searching its correlations with microbial taxa, it was mostly negatively associated with *Leptotrichia A sp000469505* and *Prevotella saccharolytica* (Supplementary Table S9), however, positively associated with *Rothia* SGBs such as *R. mucilaginosa which* was dominant in tongue dorsum and often observed in large patches toward the exterior of the consortium. The other SNP, rs73243848 located in *G2E3-AS1* was associated with salivary microbial composition (*p* = 2.35 × 10^−8^). It was mostly positively associated with *Prevotella* uSGB 2511 and family Bacteroidaceae (Supplementary Table S10).

The above analysis found that 53 host environmental factors (BH-adjusted *p* < 0.05) explained 7.78% of the β-diversity variance for salivary microbiome and 35 explained 6.36% for tongue dorsum microbiome. By applying the same number of host genetic variants as environmental factors, we found the top 53 and 35 SNPs that were most closely associated with β-diversity of the salivary and tongue dorsum microbiome explained 14.14 and 10.14% of the β-diversity variances for the two niches, respectively (Supplementary Fig. S13). The findings suggested host genetics is likely to influence the oral microbiome more than the environment.

### Host genetics and oral microbiome predict dental diseases

**Fig. 6:**
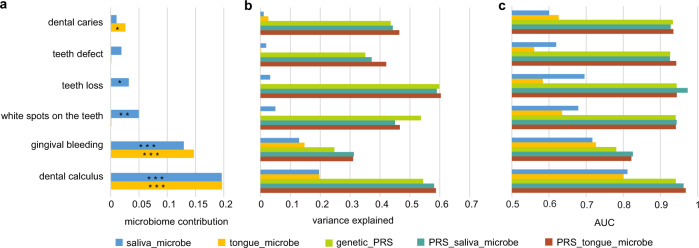
Oral microbiome and genetic PRS infer a significant fraction of the variance of dental diseases. **a** *R*^2^ estimates of six dental diseases and their significance contributed by the oral microbiome, evaluated using a linear model in the lightGBM package. *p < 0.05, **p < 0.01, and ***p < 0.001. **b** Predictive efficiency of six dental diseases (measured using the coefficient of determination (*R*^2^)), evaluated using a linear model under five different sets of predictive features: (i) relative abundances of salivary microbial taxa; (ii) relative abundances of tongue dorsum microbial taxa; (iii) PRS calculated as an unweighted sum of risk alleles from independent and significant SNPs (LD *r*^2^ < 0.2, *p* < 10^−5^) for each oral disease; (iv) “PRS + salivary microbiome”: PRS, relative abundances of salivary microbial taxa, and (v) “PRS + tongue microbiome”: PRS, relative abundances of tongue dorsum microbial taxa. **c** The discriminative efficiency for six dental diseases (measured using the area under the curve (AUC)), evaluated using a discriminative model under the five different sets of predictive features as described in **b**.

The dynamic and polymicrobial oral microbiome is a direct precursor of dental diseases such as dental caries and periodontitis^44^. To understand the aggregate effect of the host genetic variants and oral microbiome on dental diseases, we constructed models using genetic polygenic risk scores (PRS) and oral microbiome separately, as well as their combination, to predict dental diseases. We found two of the six dental diseases that occurred in over 5% of individuals to be significantly associated with the oral microbiome (Fig. 6a; FDR *p* < 0.001). Either salivary and tongue dorsum microbiome explained 20% of the variance for dental calculus. Salivary and tongue dorsum microbiome explained 13% and 15% of the variance for gingival bleeding, respectively. Compared with the oral microbiome, the genetic PRS showed significantly higher predictive efficiency with a mean *R*^2^ of 45%, ranging from the lowest of 25% for gingival bleeding to the highest of 60% for teeth loss. Furthermore, when incorporating the oral microbiome into the PRS model, the predictive efficiency is slightly improved, with a 4% increment of *R*^2^ for dental calculus and a 6% increment of *R*^2^ for gingival bleeding (Fig. 6b).

The discriminative efficiency for dental diseases was also evaluated using the area under the curve (AUC; Fig. 6c). Salivary and tongue dorsum microbiome had good discrimination for dental calculus (AUC = 0.81 and 0.80, respectively), and median discrimination for gingival bleeding (AUC = 0.72 and 0.73, respectively). The models of PRS had an AUC of 0.93–0.94 for five of the six dental diseases, except for gingival bleeding (AUC = 0.78). Incorporating the oral microbiome into the PRS model resulted in improved discrimination with AUC increasing from 0.94 to 0.97 for dental calculus and from 0.78 to 0.83 for gingival bleeding. These results may help explain why some people are genetically predisposed to major dental diseases.

## Discussion

In summary, we performed the first large-scale mgGWAS for the oral microbiome and report unequivocal human genetic determinants for the oral microbiome. Based on the metagenome-assembled profile, we identified abundant genetic loci to associate with oral microbiota. Four out of the five study-wide and nearly 1/10 genome-wide significant signals could be replicated in a low-depth genome cohort also from China, highlighting the power of adding the independent cohort for genomic association analyses of the oral microbiome. Our mgGWAS analysis found 84%–85% concordant association signals shared by the tongue dorsum and salivary microbiome, with all genome-wide significant associations in one niche (Fig. 5; *p* < 5 × 10^−8^) were also at least nominally significant in the other niche (*p* < 0.05), consistent with our and previous findings that tongue dorsum and salivary microbiome communities exhibited high levels of similarity^45,46^, especially in the micron-scale structure of oral niches^36,47^. Not only an independent cohort but also different niches (tongue dorsum and saliva) in the oral cavity corroborated the robust and replicable host-microbe association results. The non-replicated associations may require more high-depth genome cohorts or other niches microbiome for further confirmation in the future. Consistent with previous studies^23,48^, the salivary microbiome showed higher alpha diversity than tongue dorsum. In combination with the fact that saliva comes into contact with all surfaces in the oral cavity and represents a fingerprint of the general composition of the oral microbiome, these results suggested that the salivary microbiome is more diverse and likely more dynamic. Thus, host genetic associations that are stronger with the salivary than the tongue dorsum community will further invite other omics studies, especially the proteome and the nitrogen cycle that could impact microbial growth.

Host-associated microbial communities are influenced by both host genetics and environmental factors. The debate centers on the relative contribution of the host genetic and environmental factors to the human microbiome. Twins’ modeling has demonstrated that some taxa of the human oral microbiome are heritable^17,18^, however, some studies indicated oral microbiome variances were shaped primarily by the environment rather than host genetics^19,20^. With this high-depth whole-genome and metagenomic sequencing and high-quality assembled oral microbiome samples, we found that significant environmental factors explained 6.36%–7.78% of the β-diversity variance for the oral microbiome, however, the same number of significant host SNPs as environmental factors could infer 10.14%–14.14% of the β-diversity variance for oral microbiome (Supplementary Figs. S11 and S13). These findings indicated host genetics is likely to influence the oral microbiome more than the environment. A previous study identified 42 SNPs that together explained 10% of the variance of gut microbiome β-diversity^15^. The comparable explained variances of host genetic variants on both gut and oral microbiome consistently confirmed the important role of host genes in shaping the human microbiota.

There are similarities and overlaps between the oral and gut microbiome. First, the gut and oral microbial profiles showed strong observational correlations. Half (31) of the 62 prevalent salivary microbial genera (present in over 10% individuals) and 41% of the 75 prevalent tongue dorsum microbial genera significantly correlated with corresponding gut genera in abundance (Spearman *p* < 0.05) in this 4D-SZ cohort. The observed strong correlations between the two sites were supported by a metagenome-wide association study of rheumatoid arthritis^6^, although the fact that the taxonomic compositions of the two sites were different^49^. This was also consistent with the report from the HMP project revealed that oral cavity and stool bacteria overlapped in more than 45% of subjects^50^. Second, gender^49^, age^51^, geographic regions^51–53^, diets^54,55^, and serum metabolites had been reported to affect the gut microbiome composition substantially. Consistently, all these host factors also exerted significant effects on oral microbiome communities (Supplementary Tables S6 and S7). Third, the oral and gut microbiome shared some host genetic variants (Supplementary Table S11). Three of the five study-wide significant genetic loci for oral microbiome were also significantly associated with the gut microbiome. For example, the tongue dorsum microbiota-associated gene *SLC2A9* correlated with the abundance of *Bifidobacterium animalis* in the gut. The salivary microbiota-associated genetic loci *OR11H1*-*POTEH* and *FLJ36000*-*MTRNR2L1* linked to abundances of *Bacteroides fragilis* and *unclassified Enterobacter_sp._638* in the gut, respectively. Notably, the ABO blood group locus associated with the microbial module for lactose/galactose degradation in the gut^56^ was also correlated with *Solobacterium moorei* level in the tongue dorsum and *TM7x uSGB 3373* level in the saliva. Fourth, the serum uric acid was observed to significantly correlate with Lachnospiraceae species such as *Lachnoanaerobaculum umeaens*e, *uSGB 1215*, and *uSGB 489* (Fig. 3) in the tongue dorsum. Interestingly, not only in the oral cavity, the serum uric acid showed an observational correlation even a reciprocal causal relationship with fecal Lachnospiraceae species such as unclassified bacterium 9_1_43BFAA in this 4D-SZ cohort. The causal effect of serum uric acid on increased fecal unclassified bacterium 9_1_43BFAA abundance was also confirmed in the Biobank Japan cohort, as reported in our recent Mendelian randomization analyses^56^. Together, the listed pieces of evidence showed the overlaps of the oral and gut microbiome and supported the “oral–gut axis” that oral microbe transmission to and subsequent colonization of the large intestine is common and extensive among healthy individuals^3^.

As genetic characters are already there at birth, oral hygiene would be more important for people who are more likely to develop dental diseases and beyond. Despite different aetiologies, dental calculus and gingival bleeding are both driven by a combined function of the oral microbiota and host factors. However, dental caries, teeth defects, and losses were mainly determined by host genetics and less influenced by the oral microbiome in this cohort. These results help us to better understand the pathogenic mechanisms and aided the design of personalized therapeutic approaches for different oral diseases. These results also provide a rationale for repeatedly taking oral samples, to study the most stable human genome, long-term trends, and short-term dynamics in the oral microbiome.

## Materials and methods

### Study subjects

All the adult Chinese individuals in this cohort were recruited for a multi-omics study, with some volunteers providing samples from as early as 2015, which would constitute the time dimension in “4D”. The cohort included 2984 individuals with blood samples collected during a physical examination in 2017 in the city of Shenzhen, and all these individuals were enlisted for high-depth whole-genome sequencing (Supplementary Table S1). About 3932 (2017 tongue dorsum and 1915 saliva) oral samples from this cohort were newly collected for whole metagenomic sequencing from 2017 to 2018 (Supplementary Table S1). As for replication, blood samples were collected from 1494 individuals, out of which 1397 had tongue dorsum samples and 1363 had salivary samples for metagenomic sequencing. The replication cohort was designed in the same manner but organized at smaller scales in multiple cities (Wuhan, Qingdao, etc.) in China. The protocols for blood and oral collection, as well as the whole genome and metagenomic sequencing, were similar to our previous works^4,22,57^. For the blood sample, DNA was extracted using MagPure Buffy Coat DNA Midi KF Kit (no. D3537-02) according to the manufacturer’s protocol. Tongue dorsum and salivary samples were collected with an MGIEasy kit. For the salivary sample, a 2× concentration of stabilizing reagent kit was used and 2-mL saliva was collected. DNA of oral samples was extracted using MagPure Stool DNA KF Kit B (no. MD5115-02B). The DNA concentrations from blood and oral samples were estimated by Qubit (Invitrogen). About 500 ng of input DNA from blood and oral samples were used for library preparation and then processed for paired-end 100 bp sequencing using the BGISEQ-500 platform^58^.

The study was approved by the Institutional Review Boards (IRB) at BGI-Shenzhen, and all participants provided written informed consent at enrollment.

### High-depth whole-genome sequencing for the discovery cohort

About 2984 individuals with blood samples were sequenced to a mean of 33× for the whole genome. The reads were aligned to the latest reference human genome GRCh38/hg38 with BWA^59^ (v0.7.15) with default parameters. The reads consisting of base quality < 5 or containing adapter sequences were filtered out. The alignments were indexed in the BAM format using Samtools^60^ (v0.1.18) and PCR duplicates were marked for downstream filtering using Picardtools (v1.62). The Genome Analysis Toolkit’s (GATK^61^, v3.8) BaseRecalibrator created recalibration tables to screen known SNPs and INDELs in the BAM files from dbSNP (v150). GATKlite (v2.2.15) was used for subsequent base quality recalibration and removal of read pairs with improperly aligned segments as determined by Stampy. GATK’s HaplotypeCaller was used for variant discovery. GVCFs containing SNVs and INDELs from GATK HaplotypeCaller were combined (CombineGVCFs), genotyped (GenotypeGVCFs), variant score recalibrated (VariantRecalibrator), and filtered (ApplyRecalibration). During the GATK VariantRecalibrator process, we took our variants as inputs and used four standard SNP sets to train the model: (1) HapMap3.3 SNPs; (2) dbSNP build 150 SNPs; (3) 1000 Genomes Project SNPs from Omni 2.5 chip; and (4) 1000 G phase1 high confidence SNPs. The sensitivity threshold of 99.9% to SNPs and 98% to INDELs were applied for variant selection after optimizing for Transition to Transversion (TiTv) ratios using the GATK ApplyRecalibration command.

We applied a conservative inclusion threshold for variants: (i) mean depth > 8×; (ii) Hardy–Weinberg equilibrium (HWE) *p* > 10^−5^; and (iii) genotype calling rate > 98%. We demanded samples to meet these criteria: (i) mean sequencing depth > 20×; (ii) variant calling rate > 98%; (iii) no population stratification by performing principal components analysis (PCA) analysis implemented in PLINK^62^ (v1.9), and (iv) excluding related individuals by calculating pairwise identity by descent (IBD, Pi-hat threshold of 0.1875) in PLINK. No samples were removed in quality control filtering. After variant and sample quality control, 2984 individuals (out of which 2017 had matched tongue dorsum and 1915 had matched salivary samples) with about 10 million common and low-frequency (MAF ≥ 0.5%) variants were left for mgGWAS analyses.

### Low-depth whole-genome sequencing for the replication cohort

About 1494 individuals in the replication cohort were sequenced to a mean of 9× for the whole genome. We used BWA to align the whole genome reads to GRCh38/hg38 and used GATK to perform variants calling by applying the same pipelines as for the high-depth WGS data. After completing the joint calling process with CombineGVCFs and GenotypeGVCFs options, we obtained 43,402,368 raw variants. A more stringent process in the GATK VariantRecalibrator stage compared with the high-depth WGS was then used, the sensitivity threshold of 98.0% to both SNPs and INDELs was applied for variant selection after optimizing for Transition to Transversion (TiTv) ratios using the GATK ApplyRecalibration command. Further, we kept variants with less than 10% missing genotype frequency and minor allele count more than 5. All these high-quality variants were pre-phased using Eagle (v2.4.1)^63^ and then imputed using Minimac3 (v2.0.1)^64^ with our previous 1992 high-depth WGS dataset^56^ as reference panel. We retained only variants with imputation info. > 0.7, Hardy–Weinberg equilibrium *P* > 10^−5^ and genotype calling rate > 90%. Similar to what we have done for the discovery cohort, samples were demanded to have a mean sequencing depth > 5×, variant call rate > 95%, no population stratification, and no kinship. Finally, 1430 individuals (out of which 1333 had matched tongue dorsum and 1299 had matched salivary samples) with 8.6 million common and low-frequency variants (MAF ≥ 0.5%) from the replication cohort were left for association validation analysis.

### Oral metagenomic sequencing and quality control

Metagenomic sequencing was done on the BGISEQ-500 platform, with 100 bp of paired-end reads for all samples and four libraries were constructed for each lane. We generated 19.18 ± 7.90 Gb (average ± standard deviation) and 19.90 ± 7.73 Gb raw bases per sample for tongue dorsum samples in discovery and replication cohorts, respectively (Supplementary Table S1). We also generated 13.64 ± 2.91 Gb and 13.66 ± 2.80 Gb raw bases per sample for salivary samples in discovery and replication cohorts, respectively. After using the quality control module of metapi pipeline followed by reads filtering and trimming with strict filtration standards (not less than mean quality Phred score 20 and not shorter than 51 bp read length) using fastp v0.19.463, host sequences contamination removing using Bowtie2 v2.3.564 (hg38 index) and seqtk65 v1.3, we finally got an average of 13.3 Gb (host rate: 31%) and 3.1 Gb (host rate: 77%) raw bases per sample for tongue dorsum and salivary samples, respectively.

### Oral metagenomic profiling

The high-quality oral genome catalog was constructed in our previous study^22^. The oral metagenomic sequencing reads were mapped to the oral genome catalog (http://ftp.cngb.org/pub/SciRAID/Microbiome/human_oral_genomes/bowtie2_index) using Bowtie2 with parameters: “-end-to-end -very-sensitive -seed 0 -time -k 2 -no-unal -no-discordant -X 1200”, and the normalized contigs depths were obtained by using jgi_summarize_bam_contig_depths, then based on the correspondence of contigs and genome, the normalized contig depth was converted to the relative abundance of each species for each sample. Finally, we merged all representative species’ relative abundance to generate a taxonomic profile for the human oral population. The profiling workflow was implemented in the metapi jgi_profiling module (https://github.com/ohmeta/metapi/blob/dev/metapi/rules/profiling.smk#L305).

### Tongue dorsum and salivary microbiome comparison

The nonparametric Wilcoxon rank-sum test was used to determine statistically significant differences in species α-diversity between tongue dorsum and saliva niches. We analyze the β-diversity (based on genus-level Bray–Curtis dissimilarity) difference between the two oral niches using PERMANOVA (adonis) in the ‘vegan’ package and visualize the two oral niches groups using ordination such as non-metric multidimensional scaling (NMDS) plots.

### Association analysis for oral microbial taxa

After investigating the distributions of occurrence rate and relative abundance of all microbial taxa, we decided to filter the microbial taxa to keep those with occurrence rates over 90% and average relative abundance over 1 × 10^−5^. After filtering, the represented genera of these microbial taxa covered between 99.63% (tongue dorsum) and 99.76% (saliva) of the whole community in the cohort. As many oral microbial taxa are highly correlated and aim to reduce the number of GWAS tests, we then performed a number of Spearman’s correlation tests to obtain the independent taxa for mgGWAS analyses. Spearman’s correlations were calculated pairwise between all taxa, and the correlations were used to generate an adjacency matrix where correlations of > 0.8 represented an edge between taxa. A graphical representation of this matrix was then used for the greedy selection of representative taxa. Nodes (microbiota taxa) were sorted by degree and the one with the highest degree was then chosen as a final taxon (selecting at random in the case of a tie). The taxon and its connected nodes were then removed from the network and the process repeated until a final set of taxa sets were found such that each of the discarded taxa was correlated with at least one taxon. These filtering resulted in a final set of 1583 and 1685 independent microbial taxa for tongue dorsum and saliva, respectively, which were used for association analyses.

We tested the associations between host genetics and oral bacteria using a linear model based on the relative abundance of oral bacteria. Specifically, the relative abundance was transformed by the natural logarithm and the outlier individual who was located away from the mean by more than four standard deviations was removed so that the abundance of bacteria could be treated as a quantitative trait. Next, for 10 million common and low-frequency variants (MAF ≥ 0.5%) identified in this cohort, we used a linear regression model to perform mgGWAS analysis via PLINK v1.9. Given the effects of environmental factors such as diet and lifestyles on microbial features, we included all potential cofounders that were significantly associated with the β-diversity (Benjamini–Hochberg FDR < 0.05) estimates in the below explained variance analysis, as well as the top four principal components (PCs) as covariates for mgGWAS analysis in both the salivary and tongue dorsum niches. We next performed the same association analyses in the replication cohort and further validated the significance of identified associations by the discovery cohort. To investigate whether the observed number of nominally significant concordant associations with effects in the same direction was more than expected by chance, we randomly selected 455 and 466 independent associations for tongue dorsum and saliva, respectively, with ten iterations (*n* = 10). We calculated the average number of concordant replicated associations in the ten-time random iterations as expected value and then compared it to the actual replicated number using a one-sided χ^2^ test. The replication was considered significant if the χ^2^ *p* was less than 0.05.

To investigate the correlations between the identified oral microbiome-related SNPs and diseases, we downloaded the summary statistics data from the Biobank Japan^30,31^, a study of 300,000 Japanese citizens suffering from cancers, diabetes, rheumatoid arthritis, and other common diseases. We searched the oral microbiome-related SNPs in the summary statistics data from Biobank Japan to examine their associations with diseases.

### Functional and pathway enrichment analysis

The significant genetic variants identified in the association analysis were mapped to genes using ANNOVAR^65^. Given that some significant genetic variants were low-frequency in the mgGWAS results, it’s most suitable to input gene lists for enrichment analysis. We mapped variants to genes based on physical distance within a 20 kb window and got the gene lists for enrichment analysis. DAVID (https://david.ncifcrf.gov/) was utilized to perform functional and pathway enrichment analysis. DAVID is a systematic and integrative functional annotation tool for the analysis of the relevant biological annotation of gene lists and provides a functional interpretation of the GO enrichment and KEGG pathway analysis^38^. The *p* value < 0.05 was considered statistically significant. In addition, the mapped genes were further investigated using the GENE2FUNC procedure in FUMA^39^ (http://fuma.ctglab.nl/), which provides hypergeometric tests for the list of enriched mapping genes in 53 GTEx tissue-specific gene expression sets, 7246 MSigDB gene sets, and 2195 GWAS catalog gene sets^39^. Using the GENE2FUNC procedure, we examined whether the mapped genes were enriched in specific diseases or traits in the GWAS catalog as well as whether showed tissue-specific expression. Significant results were selected if a false discovery rate (FDR)-corrected *p* < 0.05 was observed.

### miRNA target prediction and bacterial growth experiments

The sequence of miR-4693-5p and miR-3977 downloaded from miRbase V22.1 (http://miRBase.org) were blasted against the genomes of *Streptococcus oralis* and *Streptococcus infantis*, respectively. The potential targets of two miRNAs were predicted by RNAhybrid^42^ depending on the minimum free energy (MFE) of secondary structure binding. The cutoff of MFE ≤ −20 kcal/mol and *p* value < 0.05 were used for selecting the final targeted genes.

The sequence of miR-3977 is 5′-GUGCUUCAUCGUAAUUAACCUUA-3′. The sequence of miR-4693-5p is 5′-AUACUGUGAAUUUCACUGUCACA-3′. They are both 23 nucleotides sequences and single-stranded linear miRNAs. All of them are synthesized by Beijing Liuhe BGI Technology Co., Ltd. *Streptococcus infantis* (storage number: ORS-AM09-O-22BH, storage date: February 10, 2016) and *Streptococcus oralis* (storage number: ORT-AF08-O-20, storage date: September 22, 2016) were stored in China National Gene Bank (CNGB). *Streptococcus orails* and *Streptococcus infantis* were cultured in presence of 2 nm/mL miRNA in the Brian Heart Infusion (BHI) medium or not. They were cultured in an anaerobic chamber at 37 °C. Strains were monitored as absorbance at 600 nm (OD600) per 2 h. The clone numbers were calculated at 2, 4, 6, and 10 h, respectively. The experiment was repeated three times in each group, and the average number of clones in each group was calculated. The results were represented by the growth curve of strains.

### Association analysis for microbiome α-diversity and β-diversity

The microbiome β-diversity (between-sample diversity) based on genus-level abundance data were generated using the “vegdist” function (Bray–Curtis dissimilarities). Then, we performed principal coordinates analysis (PCoA) based on the calculated beta-diversity dissimilarities using the “capscale” function in “vegan”. Finally, associations for β-diversity (a two-axis MDS) were performed using the manova() function from the “stats” package, in a multivariate analysis using genotypes and the same covariates stated above as variables.

### Association analysis for host environmental factors

As part of the 4D-SZ cohort, all participants in this study had records of multi-omics data, including anthropometric measurement, stool form, defecation frequency, diet, lifestyle, blood parameters, hormone, etc.^21^. A total of 423 host environmental factors are available in this cohort. Environmental metadata were first log-transformed and checked for collinearity using the Spearman correlation coefficient. Collinearity was assumed if a Spearman’s *ρ* > 0.6 or *ρ* < −0.6. Collinear variables were considered redundant and one variable from each pair was removed from further analysis, resulting in a final set of 340 variables.

To investigate the potential associations of top loci identified in microbiome GWAS with environmental variables especially for serum metabolites, we also performed GWAS analysis for the 340 environmental variables. Among the 340 environmental traits, the log_10_-transformed of the mean-normalized values was calculated for each quantitative phenotype (such as amino acids, vitamins, microelements, etc.) and a linear regression model for the quantitative trait implemented in the PLINK v1.9 was used for association analysis. Samples with missing values and values beyond 4 s.d. from the mean were excluded from the association analysis. For each binary phenotype (such as diet, lifestyle, etc.), a logistic regression model was used for association analysis. Age, gender, and the top four PCs were included as covariates for each association analysis.

### Environmental factors explained the variance of the oral microbiome

We next searched for associations between the 340 environmental variables selected above and the oral microbiome compositions. We performed Bray–Curtis distance-based redundancy analysis (dbRDA) to identify variables that are significantly associated with β-diversity and measure the fraction of variance explained by the factors, using the “capscale” function in the vegan package. The significance of each response variable was confirmed with an analysis of variance (ANOVA) for the dbRDA (anova.cca() function in the vegan package). Only the variables that were significantly associated (Benjamini–Hochberg FDR < 0.05) with the β-diversity estimates in the univariable models were included in the multivariable model. The additive explanatory value (in %) of significant response variables (e.g., environmental parameters, vitamins, serum amino acids, etc.) was assessed with a variation partitioning analysis of the vegan package (“adj.r.squared” value using RsquareAdj option).

### Construct PRS for diseases prediction

To obtain the predictions of human genetics on dental diseases, we used gradient boosting decision trees from the LightGBM (v.3.1.1) package^66^ implemented in Python (v3.7.8) and a fivefold cross-validation scheme to construct risk-prediction models. In every fold of the fivefold cross-validation scheme, we calculated the associations between SNPs and dental diseases within the training dataset, and then selected independent and significant SNPs (LD *r*^2^ < 0.2, *p* < 10^−5^) to calculate the PRS as an unweighted sum of risk alleles, and finally we trained a model on the PRS and predicted the disease risk in the test dataset. During the process, we obtained the optimal values of the tuning parameters using fivefold cross-validation and evaluated the results using the coefficient of determination (*R*^2^) as variance explained and AUC as disease discriminative efficiency.

## Supplementary information

Supplementary Table S1

Supplementary Table S2

Supplementary Table S3

Supplementary Table S4

Supplementary Table S5

Supplementary Table S6

Supplementary Table S7

Supplementary Table S8

Supplementary Table S9

Supplementary Table S10

Supplementary Table S11

Supplementary Figures

## Data Availability

All summary statistics that support the findings of this study including the associations between host genetics and tongue dorsum microbiome, host genetics, and saliva microbiome are publicly available from https://db.cngb.org/search/project/CNP0001664. The release of these summary statistics data was approved by the Ministry of Science and Technology of China (Project ID: 2021BAT1539). According to the Human Genetic Resources Administration of China regulation and the institutional review board of BGI-Shenzhen related to protecting individual privacy, sequencing data are controlled-access and are available via the application on request (https://db.cngb.org/search/project/CNP0001664).
