# Supplementary material for: Metagenome-genome-wide association studies reveal human genetic impact on the oral microbiome
Source: Cell Discov. 2021 Dec 7;7:117. doi: 10.1038/s41421-021-00356-0 (PMC8648780; doi:10.1038/s41421-021-00356-0)
Supplement: Supplementary file 12 — Supplementary Figures [file 41421_2021_356_MOESM12_ESM.pdf]

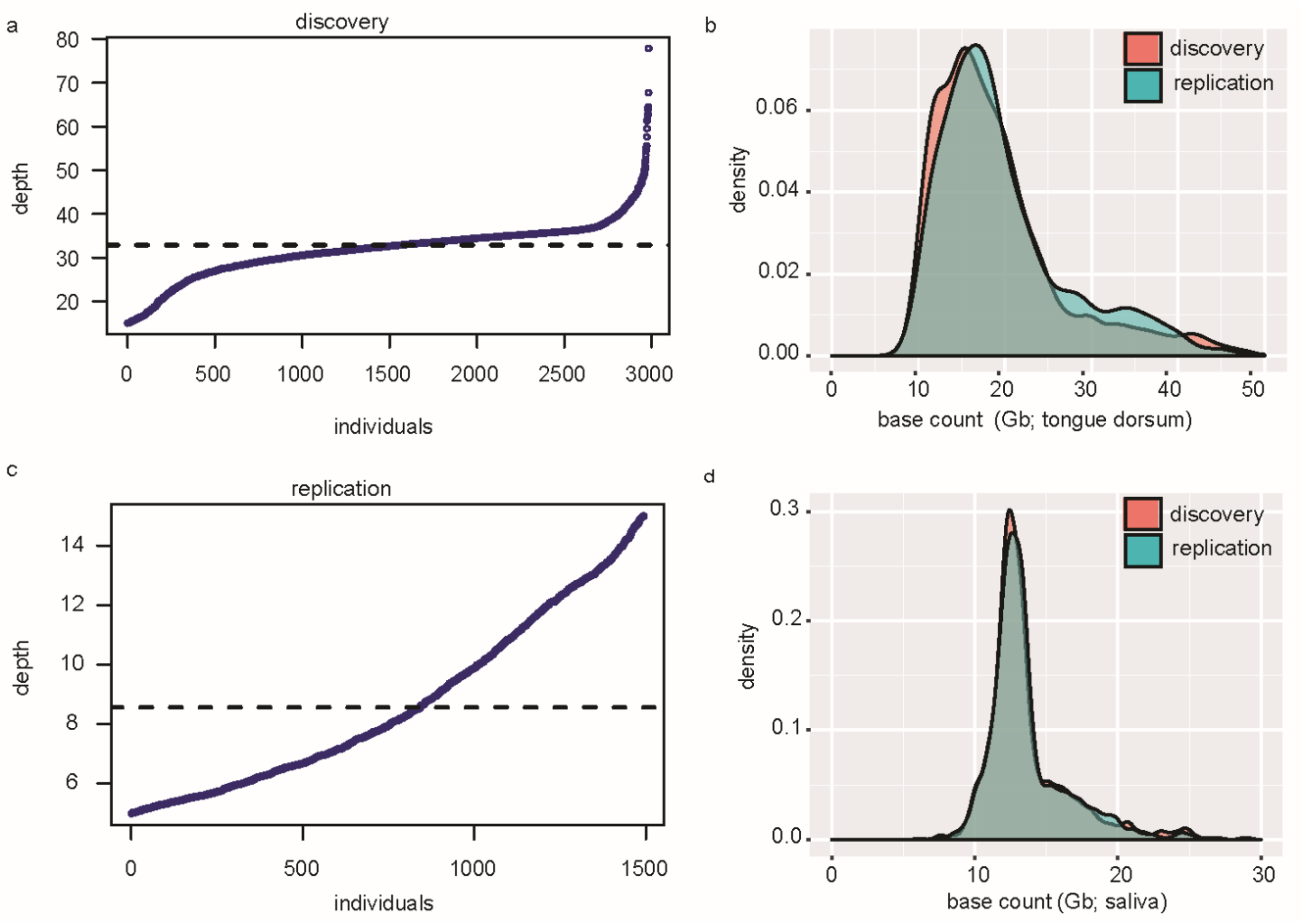

Supplementary Fig. S1. Whole-genome sequencing (WGS) and metagenome sequencing data production. (**a**) Depth distribution of 2,984 high-depth WGS samples in discovery cohort. The mean depth is 33× (ranging from 15× to 78×). (**b**) Metagenome sequencing at an average of 19.18 ± 7.90 Gb and 19.90 ± 7.73 Gb in discovery and replication cohort, respectively, for tongue dorsum samples. (**c**) Depth distribution of 1,494 low-depth WGS samples in replication cohort. The mean depth is 9× (ranging from 5× to 15×). (**d**) Metagenome sequencing at an average of 13.64 ± 2.91 Gb and 13.66 ± 2.80 in discovery and replication cohort, respectively, for salivary samples.

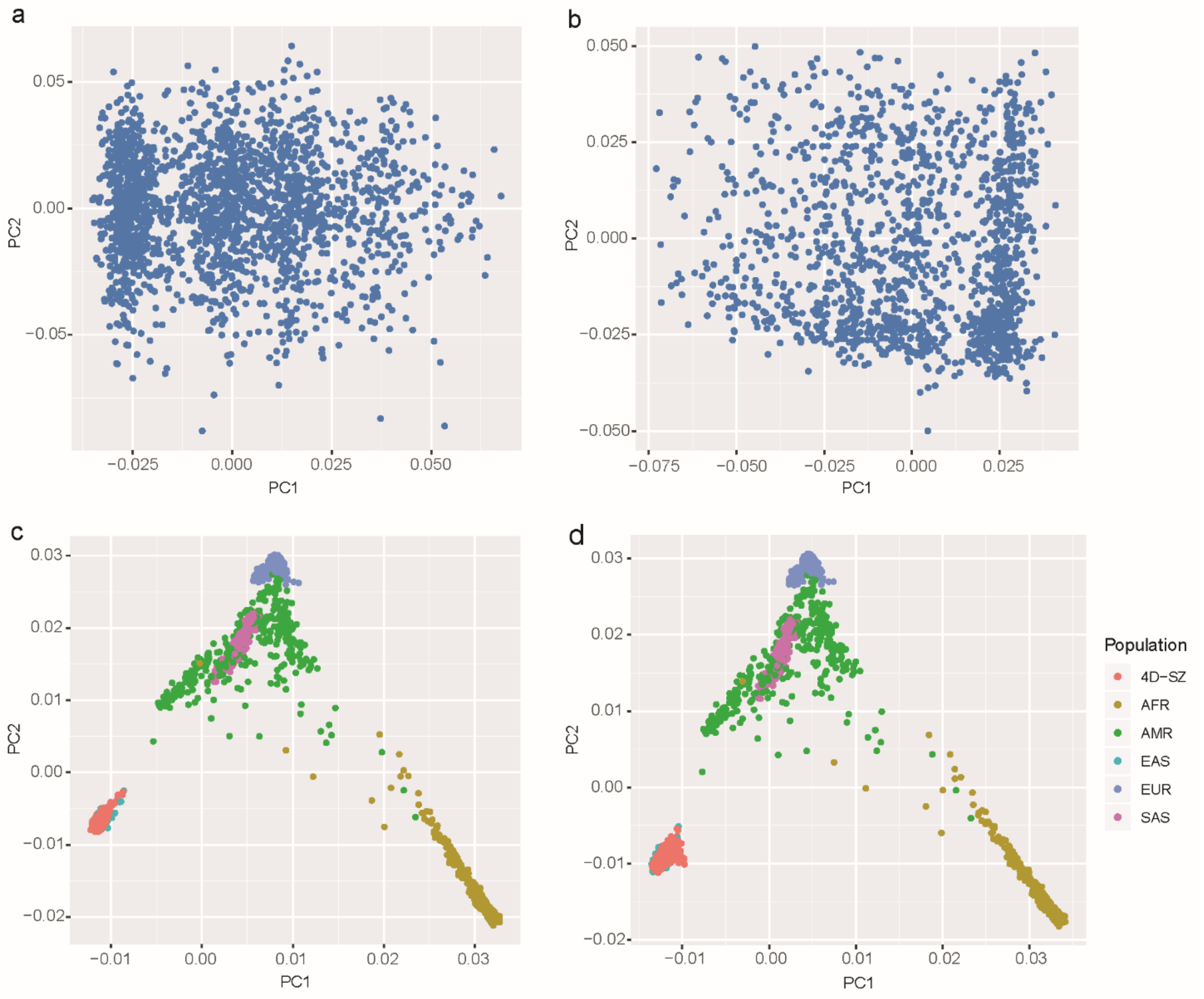

Supplementary Fig. S2. PCA plots of this 4D-SZ cohort and 1000genome phase3 population. (**a**) PCA plot of 2,984 high-depth WGS samples in discovery cohort. (**b**) PCA plot of 1,494 low-depth WGS samples in replication cohort. (**c**) PCA plot of this discovery cohort and 1000genome phase3 population. (**d**) PCA plot of this replication cohort and 1000genome phase3 population.

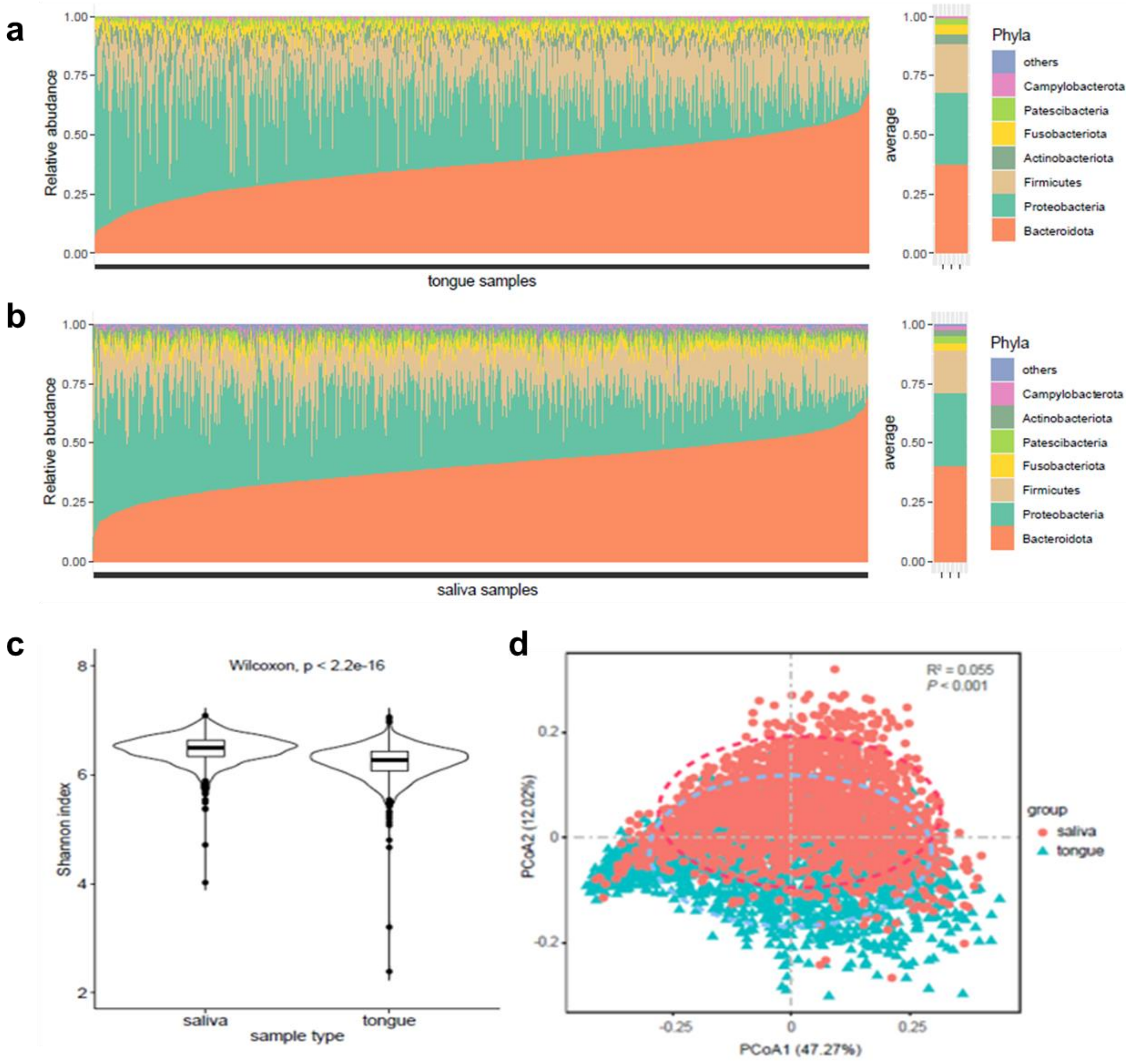

Supplementary Fig. S3. Diversity of oral microbiome in saliva and tongue dorsum samples. (**a-b**). Relative abundance of the seven main bacterial phyla found in both tongue dorsum (**a**) and saliva samples (**b**). (**c**). Alpha-diversity (Shannon index) of microbiome across the two oral sites, with the saliva presenting higher diversity in relation to the tongue dorsum. (**d**). Ordination of salivary and tongue dorsum samples based on genus-level Bray–Curtis dissimilarity using principal coordinate analysis. The first and second axes explaining the highest amount of variance, PCoA1 and PCoA2, are shown. Dashed ellipses represent the 95% confidence level of permutational multivariate analysis of variance (PERMANOVA) test. Explained variance (R^2) and p-value were also been showed.

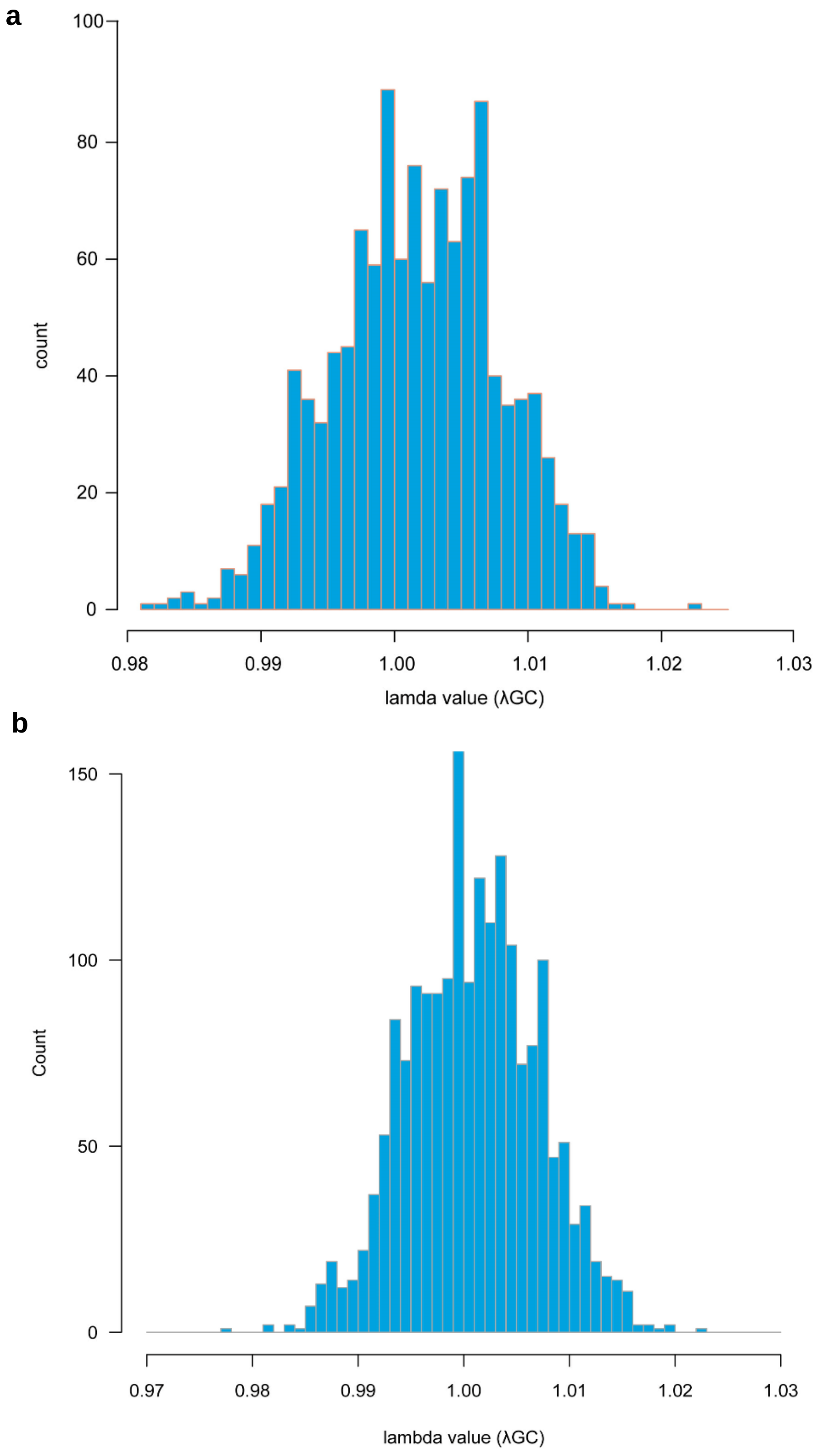

Supplementary Fig. S4. Distribution of the lambda value (λ_{GC}). (**a**) The 1,583 independent M-GWAS tests on tongue dorsum microbiome (λ_{GC} ranged from 0.981 to 1.023 with median 1.005) and (**b**) 1,685 independent M-GWAS tests on salivary microbiome (λ_{GC} ranged from 0.978 to 1.022 with median 1.002).

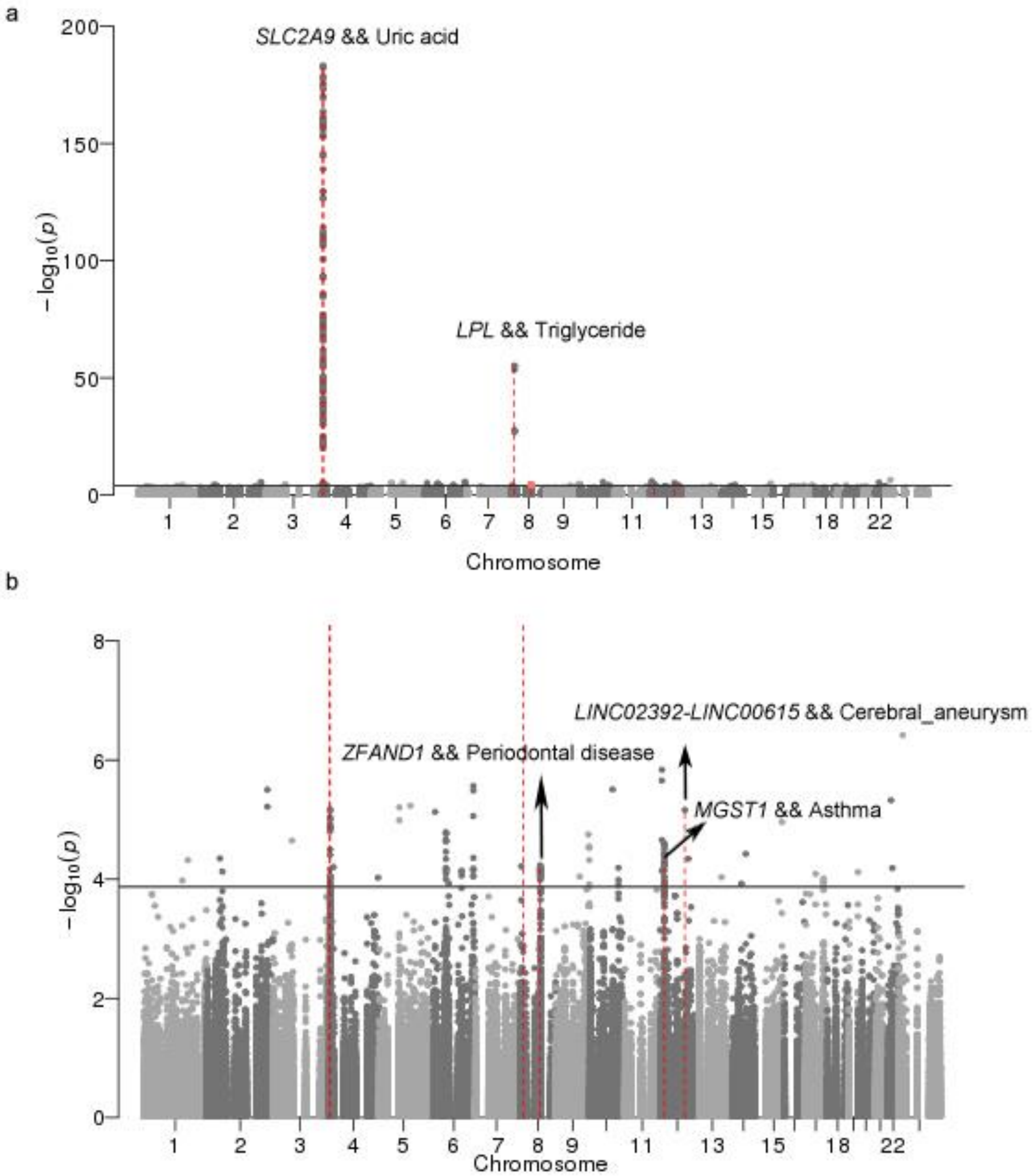

Supplementary Fig. S5. Manhattan plot shows the association results of top signals identified by tongue M-GWAS with diseases from Biobank Japan and metabolites in this cohort. *SLC2A9* that associated with *Oribacterium uSGB 1215* linked to low uric acid level. *LPL* that associated with *Haemophilus D parainfluenzae A* linked to triglyceride concentration. *MGST1* that associated with *Streptococcus uSGB 2460* linked to asthma. (**a**) represents the whole Manhattan plot and (**b**) represents the part of Manhattan plot which zooms the association results between 1 and 5×10^{-8} of p-value. The grey line represented the multiple test *p* threshold of 1.34×10^{-4} (=0.05/372 for 372 diseases or traits).

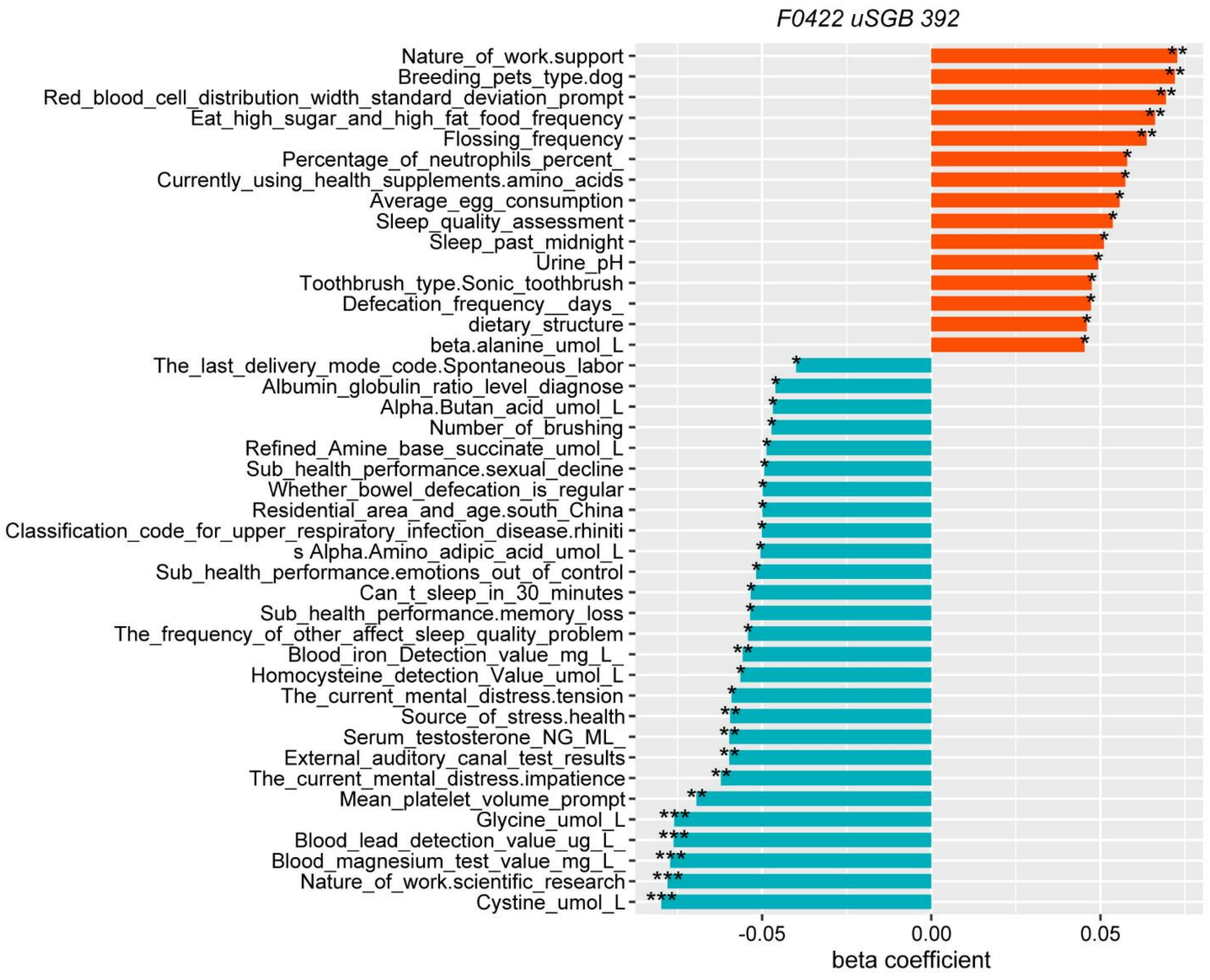

Supplementary Fig. S6. The correlation of species *F0422 uSGB 392* (belonging to family Veillonellaceae) with phenotype traits in this cohort. The linear regression model was used with adjusting sex, age and the top four principal components (PC1, PC2, PC3 and PC4). Only correlations with p<0.05 were showed. Significant code: 0.05 * 0.01 ** 0.001 *** <0.001.

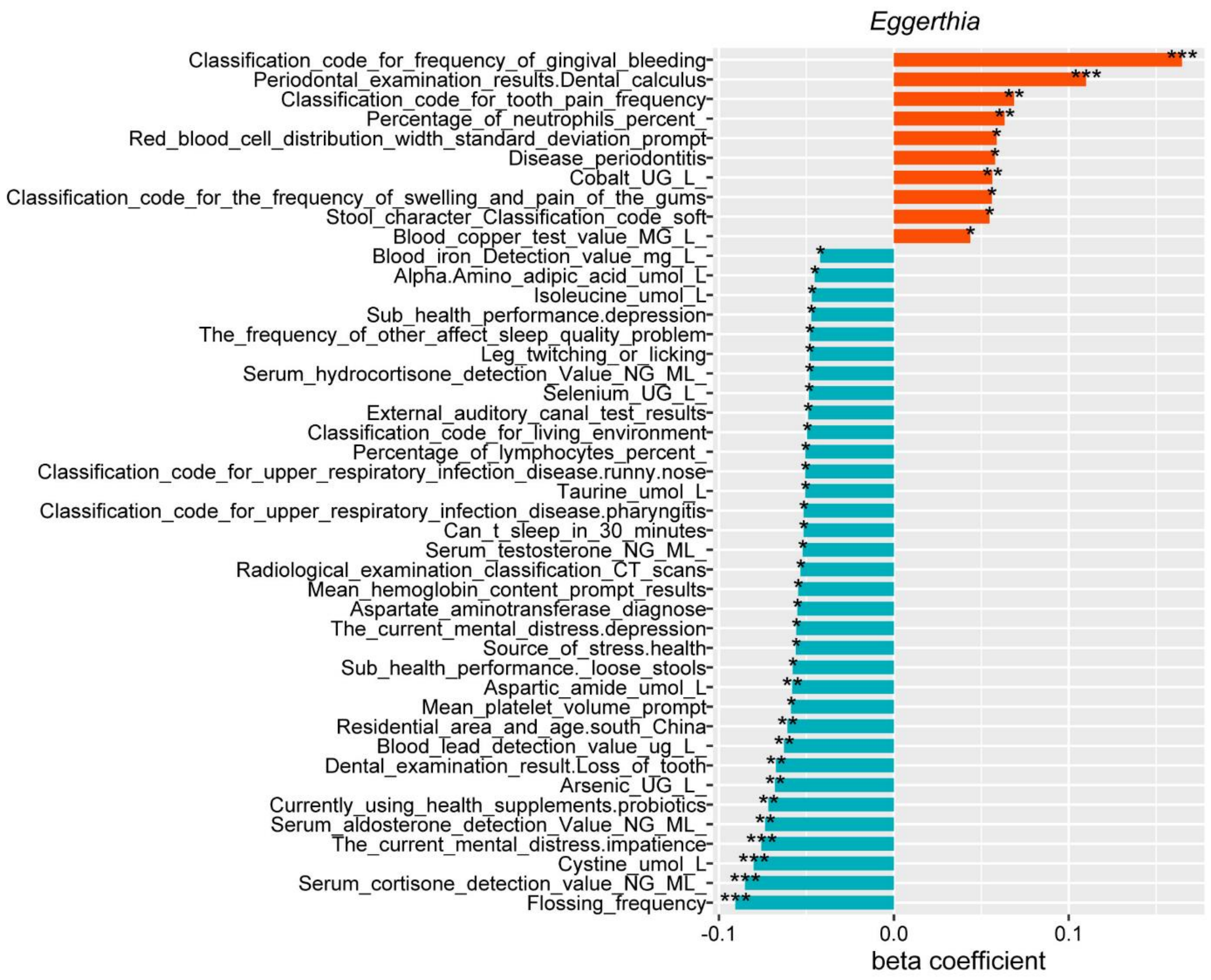

Supplementary Fig. S7. The correlation of genus *Eggerthia* with phenotype traits in this cohort. The linear regression model was used with adjusting sex, age and the top four principal components (PC1, PC2, PC3 and PC4). Only correlations with p<0.05 were showed. Significant code: 0.05 * 0.01 ** 0.001 *** <0.001.

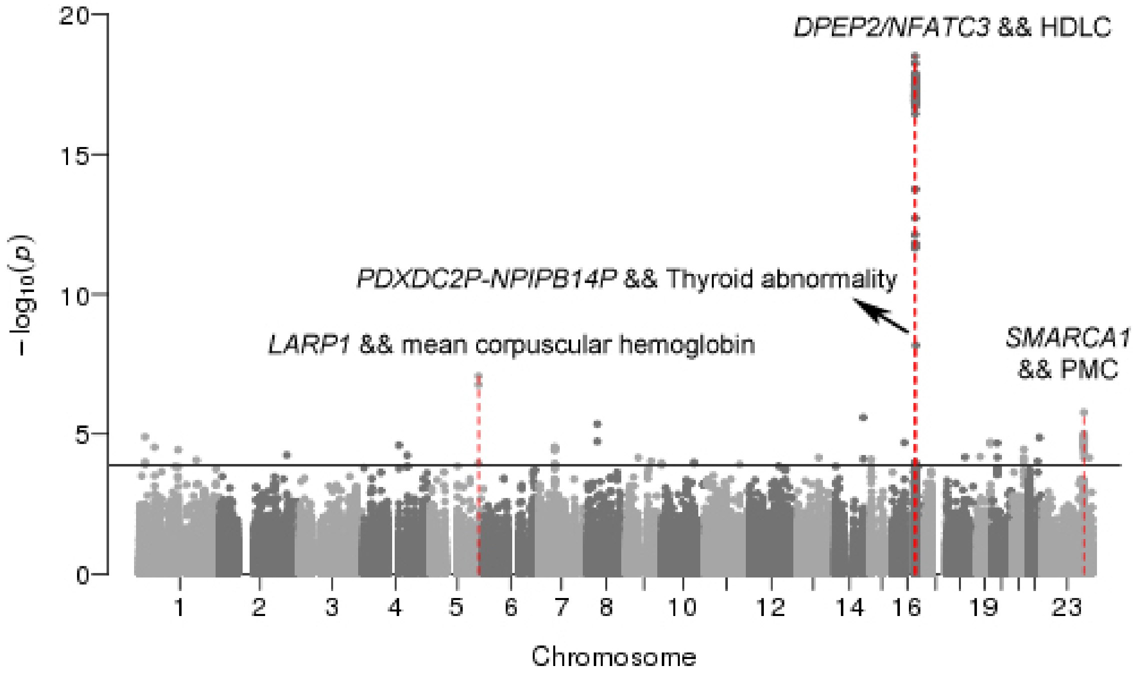

Supplementary Fig. S8. Manhattan plot shows the association results of top signals identified by saliva M-GWAS with diseases from Biobank Japan and metabolites in this cohort. *DPEP2/NFATC3* that associated with species *Lancefieldella sp000564995* linked to high density lipoprotein cholesterol (HDLC). *PDXDC2P-NPIPB14P* associated with species *Centipeda sp000468035* linked to thyroid abnormality. *LARP1* associated with species *Aggregatibacter kilianii* linked to mean corpuscular hemoglobin. *SMARCA1* associated with species *Veillonella parvula* linked to pharyngeal mucosal congestion (PMC). The grey line represented the multiple test *p* threshold of 1.34×10^{-4} (=0.05/372 for 372 diseases or traits).

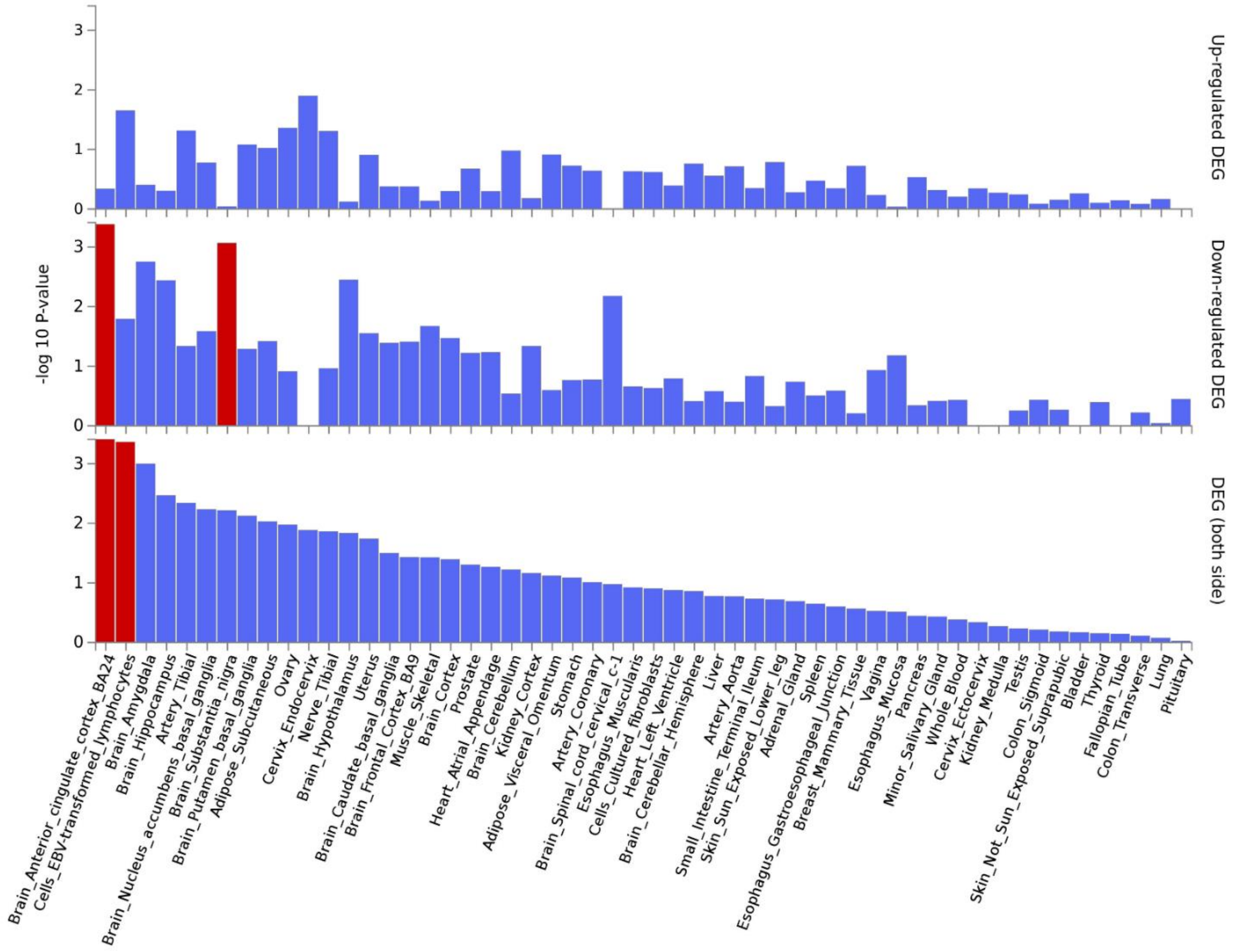

Supplementary Fig. S9. Significantly enriched differentially expressed gene (DEG) sets ($p_{adjusted}$ < 0.05) of top signals from saliva M-GWAS analysis. The significant loci with $p < 5 \times 10^{-8}$ identified in salivary M-GWAS analysis were mapped to genes based on physical distance within a 20kb window. Mapped genes were further investigated using the GENE2FUNC procedure in FUMA (http://fuma.ctglab.nl/), which provides hypergeometric tests of enrichment of the list of mapped genes in 53 GTEx tissue-specific gene expression sets.

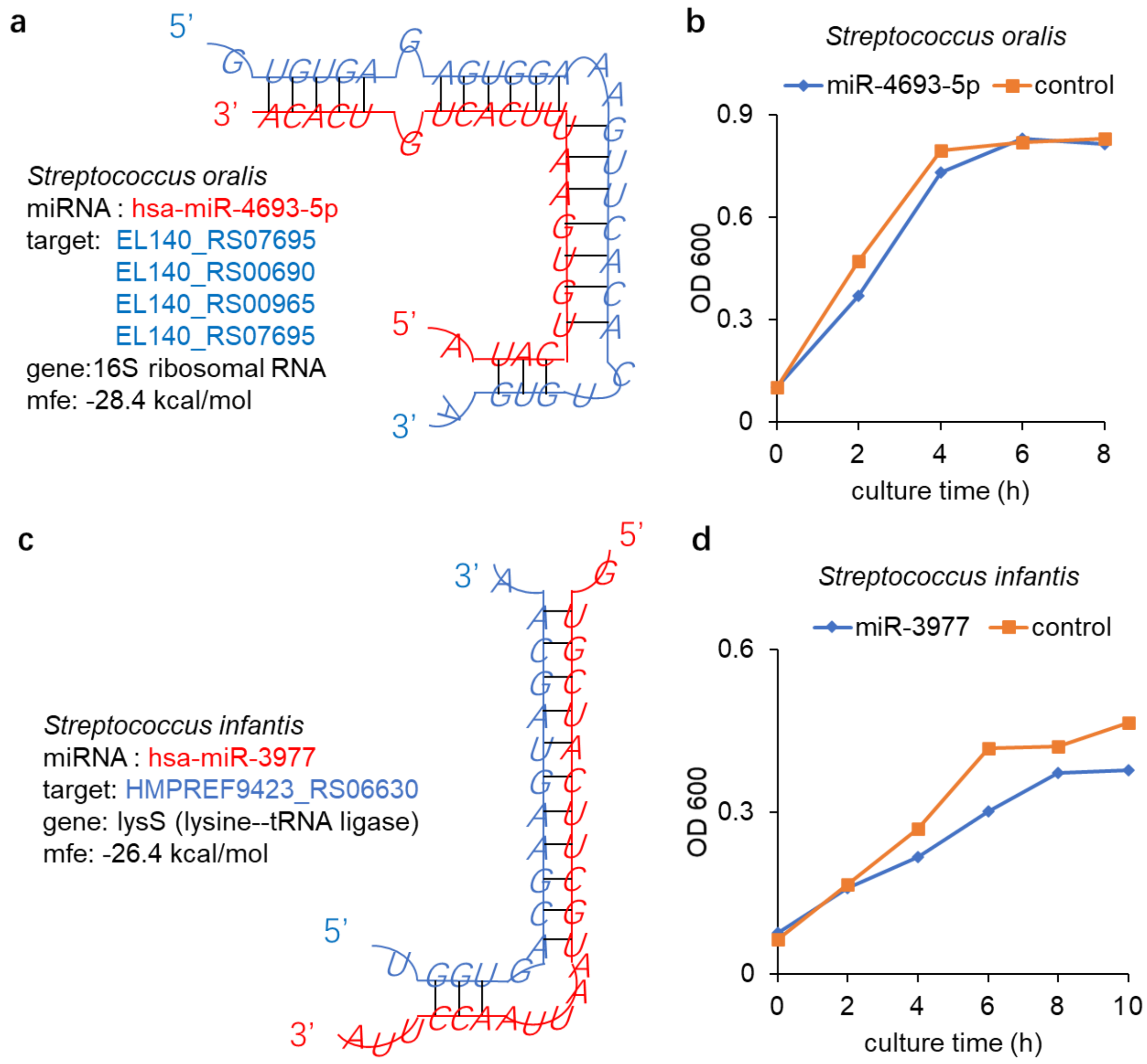

Supplementary Fig. S10. Prediction of host miRNAs targeting bacterial genes and their effects on *Streptococcus* bacterial growth. (**a**) *S. orails* genes (EL140_RS07695, EL140_RS00690, EL140_RS00965, EL140_RS07695) were predicted to be targeted by miR-4693-5p via sequence blast and predicted for secondary structure property with minimum mfe (minimum free energy) by RNAhybrid. (**b**) Growth curve of *S. oralis* cultured in the presence of miR-4693-5p or not. (**c**) *S. infantis* gene HMPREF9423_RS06630 (lysS; lysine--tRNA ligase) were predicted to be targeted by miR-3977 with minimum mfe by RNAhybrid. (**d**) Growth curve of *S. infantis* cultured in the presence of miR-3977 or not.

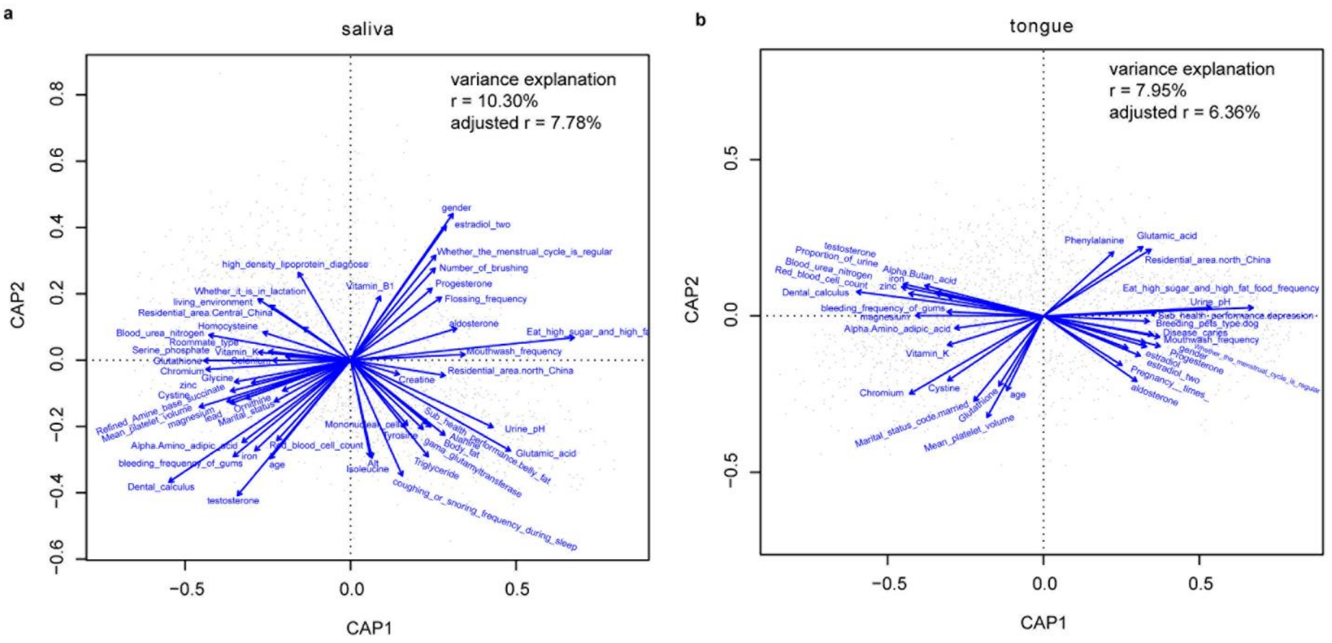

Supplementary Fig. S11. Significant environmental factors contributed to salivary (a) and tongue dorsum (b) microbiome composition (BH-adjusted FDR < 0.05).

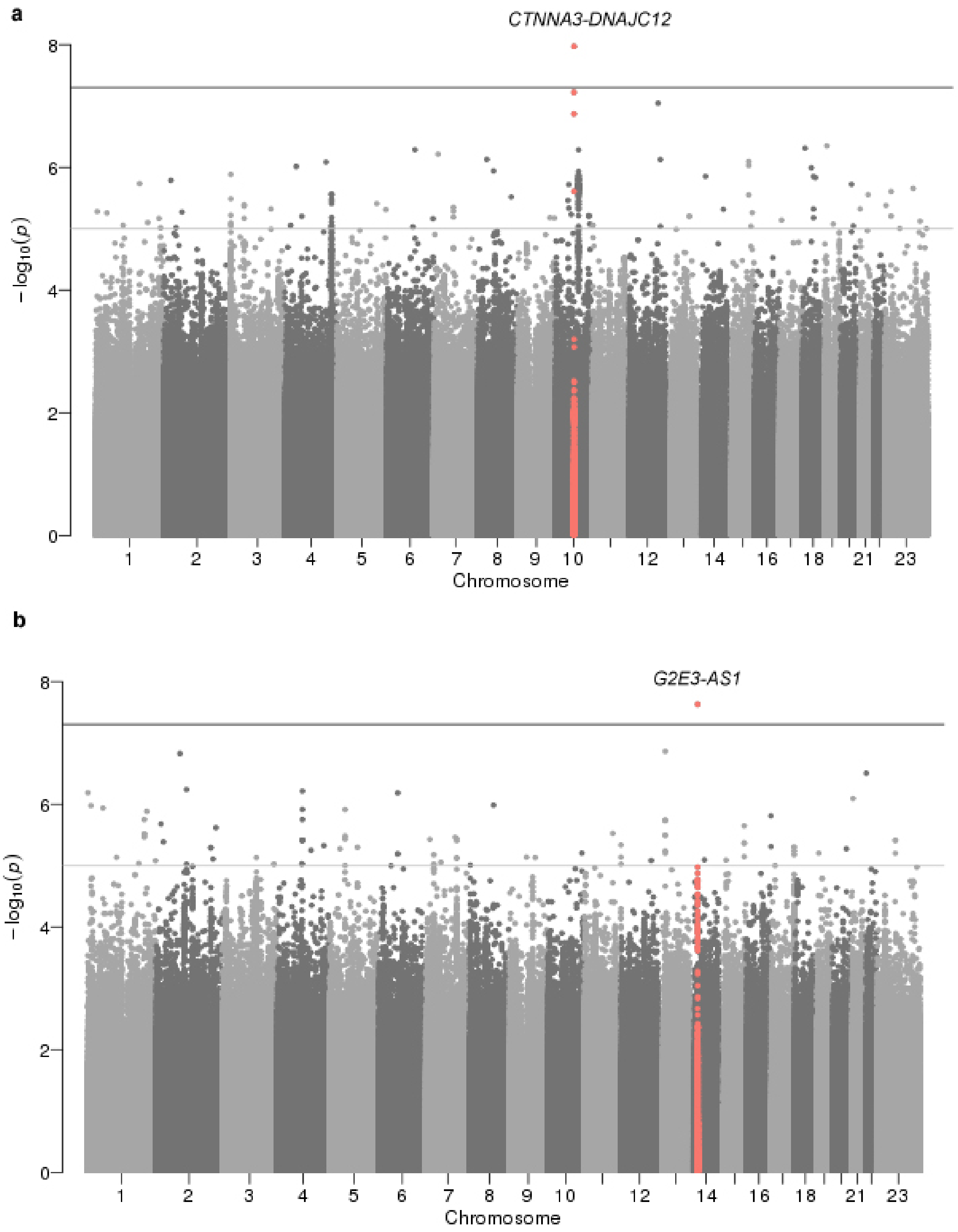

Supplementary Fig. S12. Host genetic associations with community compositions (beta-diversity) of tongue dorsum (a) and salivary (b) microbiome, respectively.

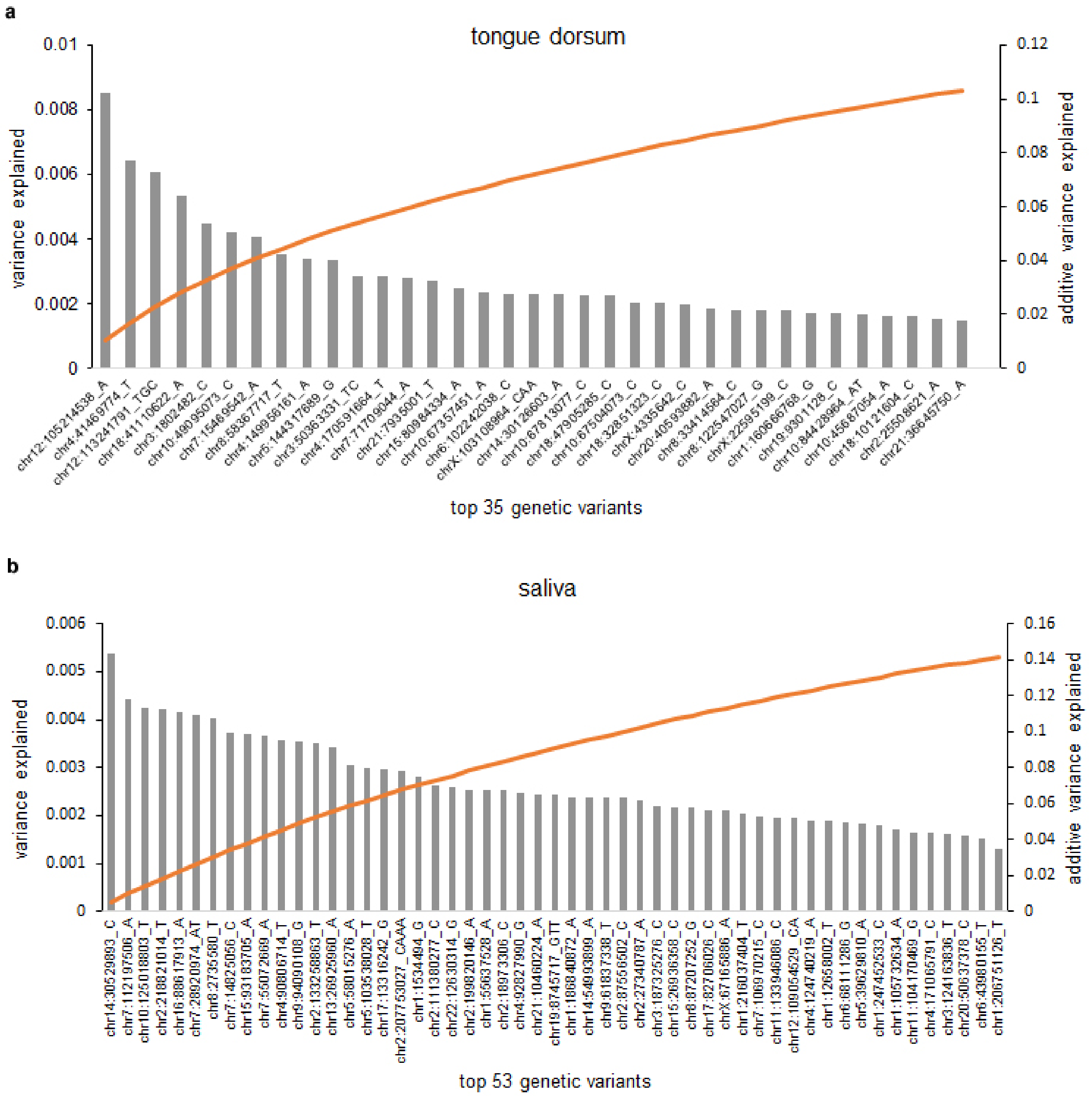

Supplementary Fig. S13. Variance explained by top genetic variants for community compositions of tongue dorsum (a) and salivary (b) microbiome, respectively.
